# ROIMCR: a powerful analysis strategy for LC-MS metabolomic datasets

**DOI:** 10.1186/s12859-019-2848-8

**Published:** 2019-05-17

**Authors:** Eva Gorrochategui, Joaquim Jaumot, Romà Tauler

**Affiliations:** Department of Environmental Chemistry, Institute of Environmental Assessment and Water Research (IDAEA), Consejo Superior de Investigaciones Científicas (CSIC), Jorsi Girona 18-25, Barcelona, 08034 Catalonia Spain

**Keywords:** LC-MS, Data analysis, Data compression, Data resolution, Regions of interest (ROI), MCR-ALS, Metabolomics, Lipidomics, Chemometrics, Untargeted analysis

## Abstract

**Background:**

The analysis of LC-MS metabolomic datasets appears to be a challenging task in a wide range of disciplines since it demands the highly extensive processing of a vast amount of data. Different LC-MS data analysis packages have been developed in the last few years to facilitate this analysis. However, most of these strategies involve chromatographic alignment and peak shaping and often associate each “feature” (i.e., chromatographic peak) with a unique m/z measurement. Thus, the development of an alternative data analysis strategy that is applicable to most types of MS datasets and properly addresses these issues is still a challenge in the metabolomics field.

**Results:**

Here, we present an alternative approach called ROIMCR to: i) filter and compress massive LC-MS datasets while transforming their original structure into a data matrix of features without losing relevant information through the search of regions of interest (ROIs) in the m/z domain and ii) resolve compressed data to identify their contributing pure components without previous alignment or peak shaping by applying a Multivariate Curve Resolution-Alternating Least Squares (MCR-ALS) analysis. In this study, the basics of the ROIMCR method are presented in detail and a detailed description of its implementation is also provided. Data were analyzed using the MATLAB (The MathWorks, Inc., www.mathworks.com) programming and computing environment. The application of the ROIMCR methodology is described in detail, with an example of LC-MS data generated in a lipidomic study and with other examples of recent applications.

**Conclusions:**

The methodology presented here combines the benefits of data filtering and compression based on the searching of ROI features, without the loss of spectral accuracy. The method has the benefits of the application of the powerful MCR-ALS data resolution method without the necessity of performing chromatographic peak alignment or modelling. The presented method is a powerful alternative to other existing data analysis approaches that do not use the MCR-ALS method to resolve LC-MS data. The ROIMCR method also represents an improved strategy compared to the direct applications of the MCR-ALS method that use less-powerful data compression strategies such as binning and windowing. Overall, the strategy presented here confirms the usefulness of the ROIMCR chemometrics method for analyzing LC-MS untargeted metabolomics data.

**Electronic supplementary material:**

The online version of this article (10.1186/s12859-019-2848-8) contains supplementary material, which is available to authorized users.

## Background

The challenge of analyzing data is one of the main concerns of metabolomic liquid chromatography coupled to mass spectrometry (LC-MS) studies [1]. Several software packages exist for MS-based metabolomic data analysis, including proprietary commercial, open-source, and online workflows [2]. Some commercial tools provided by major vendors of MS and omics high throughput analytical instruments and equipment include MassHunter (Agilent Technologies), SIEVE (Thermo Scientific) and Progenesis QI (Waters). Some of the most frequently used open-source software packages include XCMS [3, 4] (and XCMS-based Metabox [5], metaX [6]), CAMERA [7], MAIT [8], MetaboAnalyst [9], Workflow4Metabolomics [10], MZmine [11] and MetAlign [12]. However, none of these approaches are highlighted as the best strategy, and the analysis of LC-MS data remains an unresolved problem in the bioinformatics field due to the methodological discrepancies existing among these approaches.

The analysis of high-resolution LC-MS-based metabolomic datasets usually begins with filtering and compression, which is required to reduce their size into formats that are manageable with computers (without compromising the original information) and prevent errors linked to the restricted memory capacity of the computers. In addition to compressing data, in this first step, the conversion of raw data into a matrix representation is also required to obtain a set of well-structured variables (features) to analyze. The generated data matrices (x, y) are arranged with retention times in the rows (x-direction) and m/z values in the columns (y-direction). A classical procedure used for data compression and matrix transformation is binning. Using the binning procedure, high-resolution raw mass spectra are converted into a matrix representation by dividing the m/z axis into parts with a specific bin size that is generally set to a multiple of the mass accuracy of the mass spectrometer. However, a significant disadvantage of binning is the complication related to the proper choice of the bin size for a specific dataset, and the selection of the m/z bin size strongly correlates with the recovery of the proper elution profile peak shape. If the selected bin size is excessively small, chromatographic peaks fluctuate between bins and therefore are unable to be determined because of the chromatographic shape of the peak is not visible. If the bin size is excessively large, various peaks may occur in the same bin, and tiny peaks might disappear due to the elevated noise level [13]. Moreover, peak splitting might occur for equidistant binning, regardless of the bin size. One major drawback of binning is the reduction in spectral accuracy originating from the compression of data in the m/z-mode dimension, which hinders the final identification of metabolites. Moreover, in most cases, the compression performed with binning is not sufficient and further windowing (i.e., independently selecting continuous regions in the rows (time) or the columns (m/z) to be analyzed) is necessary. Nevertheless, when performing windowing, the whole process is more tedious and time-consuming, since one sample must be analyzed in several parts.

A better alternative strategy to binning and windowing is based on the idea of assuming that analyte signals are a domain of data points with a high density arranged in a particular “data void”, as first presented by Stolt et al. [14] These regions where analytes are found are called regions of interest (ROIs) and are searched according to specific criteria (i.e., a particular threshold intensity, admissible mass error and minimum number of occurrences). Overall, the ROI strategy consists of considering data included in these regions while rejecting the other data. This strategy has already been implemented in the centWave algorithm of XCMS software [13]. The result of the search for ROIs in a sample is a set of mass traces with distinct dimensions that must ultimately be reorganized into a data matrix. In contrast to the binning procedure, no reduction in spectral resolution occurs as a result of the application of the ROI searching procedure, since the bin size is not fixed. Thus, the ROI strategy allows researchers to take full advantage of all the benefits of high-resolution MS techniques. Currently, many of the current metabolomic data analysis software tools use ROI compression as a preliminary step for peak detection and/or integration.

Following the ROI search, data filtering and compression, the next crucial step in LC-MS-based metabolomic data analysis is data resolution. Most of the existing LC-MS data analysis approaches require two steps (i.e., chromatographic peak modelling and alignment) before peak resolution. Alignment methods search for matching peaks over various chromatographic runs and peak modelling methods force peaks to have a delimited and more regular shape, typically through the application of continuous wavelet transformations (CWT) and optional Gaussian fitting [15]. Therefore, preliminary peak modelling and alignment appear as an indispensable step in most of the currently available data analysis packages and are often linked to an unknown amount of sources of error. In contrast, neither of the two corrections (i.e., peak modelling and alignment) are required when using Multivariate Curve Resolution-Alternating Least Squares (MCR-ALS) [3] methods, since no modelling of elution profiles (peaks) is required (see below) and the aligned data are only produced in the spectral direction or mode. MCR methods are particularly powerful for mixture analysis and resolution in the simultaneous analysis of multirun chromatographic data.

The main goal of MCR-ALS methods is to resolve spectra arising from mixtures of the chemical constituents present in a sample into contributions from the individual components in the mixtures. Namely, MCR-ALS seeks to model the underlying physical processes that generate the data in terms of the composition of a sample. MCR-ALS-resolved MS spectra profiles are then immediately used to identify the chemical identities of metabolites through a comparison with standards or by searching a library. In the last few years, MCR-ALS methods have emerged as highly effective tools to resolve the lack of instrumental selectivity and coelution problems in different application areas, particularly in LC-MS-based metabolomic datasets.

**Fig. 1:**
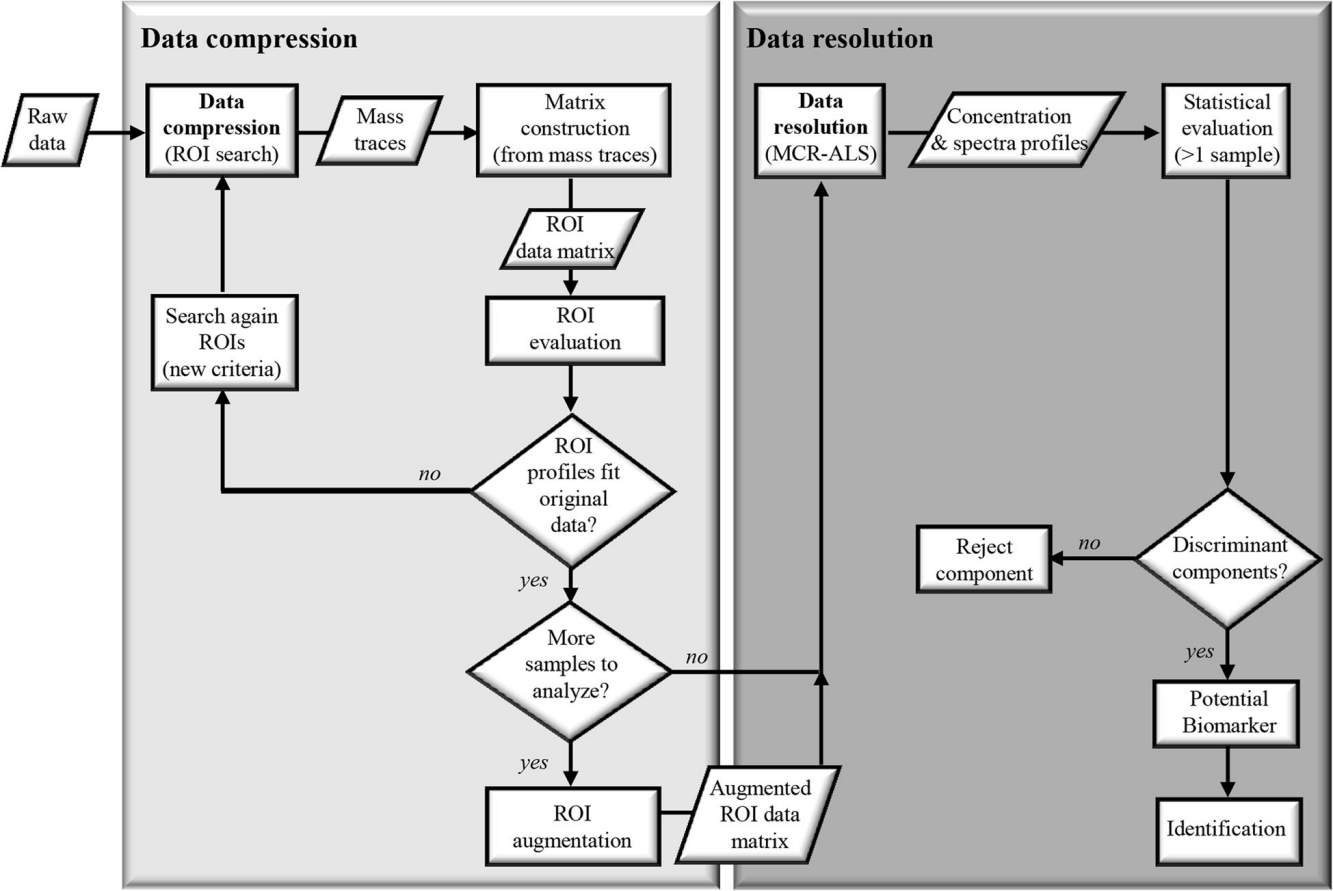
Schematic representation of the different stages of the ROIMCR approach. Initially, raw data are filtered and compressed through the search of regions of interest (ROI) and the obtained mass traces are reorganized into a matrix representation. Then, ROI profiles are evaluated: if they do not fit original data, the ROI search is repeated but changing initial criteria; on the contrary, if they properly fit original data the obtained ROI matrix is resolved by MCR-ALS. When having more than one sample, following individual ROI searches, column-wise augmented ROI data matrices can be generated and finally analyzed by MCR-ALS. Results of MCR-ALS analysis can be subsequently evaluated by statistical tests to find more significant components in the differentiation among sample groups (i.e., stressed groups vs. control groups)

In this study, we describe a new data analysis strategy, ROIMCR, designed to filter, compress and resolve LC-MS metabolomic datasets. Data filtering and compression are performed without losing spectral accuracy by searching ROIs, and chromatographic elution profiles (peaks) are resolved through the application of an MCR-ALS analysis. The main steps involved in data compression and data resolution are presented in Fig. 1. As shown in the figure, after a first data compression step through the search of ROIs, the obtained profiles are evaluated to determine whether they properly agree with original data features. ROI searching is performed on a single LC-MS sample (one dataset) or on multiple LC-MS samples (multiple datasets), generating column-wise augmented ROI data matrices in the latter case (i.e., matrices containing distinct submatrices related to distinct samples attached sequentially). The generated augmented ROI matrices are further analyzed using MCR-ALS. Finally, the ultimate step is the statistical evaluation of the resolved MCR-ALS components to discover potential biomarkers. A distinct feature of the proposed ROIMCR strategy is its current implementation in the powerful MATLAB computing and visualization environment, which is frequently used in the chemometrics field and in scientific and technological software development with all its advantages and large number of toolboxes already incorporated.

Moreover, in this study, we provide an example of the performance of the ROIMCR strategy on analyzing a lipidomic LC-MS dataset. The illustrated lipidomic data set was generated in an experiment performed in a previous study by the authors [16] in which a human placental chroriocarcinoma cell line (JEG-3) was exposed to the endocrine disruptor chemical tributyltin (TBT). Examples of other recent applications to more complex systems have been recently published [17–22] and are briefly described in “Applications of the ROIMCR procedure” section of this manuscript. Researchers interested in the ROIMCR procedure can test this strategy using the example data and the MATLAB functions for ROI compression, both of which are provided in a protocol written by the authors [22]. That protocol, which is available at https://www.nature.com/protocolexchange/protocols/4347, provides a step-by-step description of the implementation of the ROIMCR procedure. In the present study, a detailed description of the basics and fundamentals of the methodology is presented.

## Methods

A description of the ROI methodology is provided here. In addition, a brief description of the MCR-ALS method is presented below to facilitate the understanding of the whole ROIMCR procedure. MCR-ALS solves the MCR bilinear model (see Eqs. (1) and (2) below) using an alternating least squares optimization algorithm. The MCR-ALS method is already a well-stablished chemometric method and its principles and basis have been described in previous studies [23–25]. Its software implementation in the MATLAB computing and visualization language (The MathWorks Inc., https://www.mathworks.com) and other details are found on its official webpage: www.mcrals.info.

### ROI search in one LC-MS sample

**Fig. 2:**
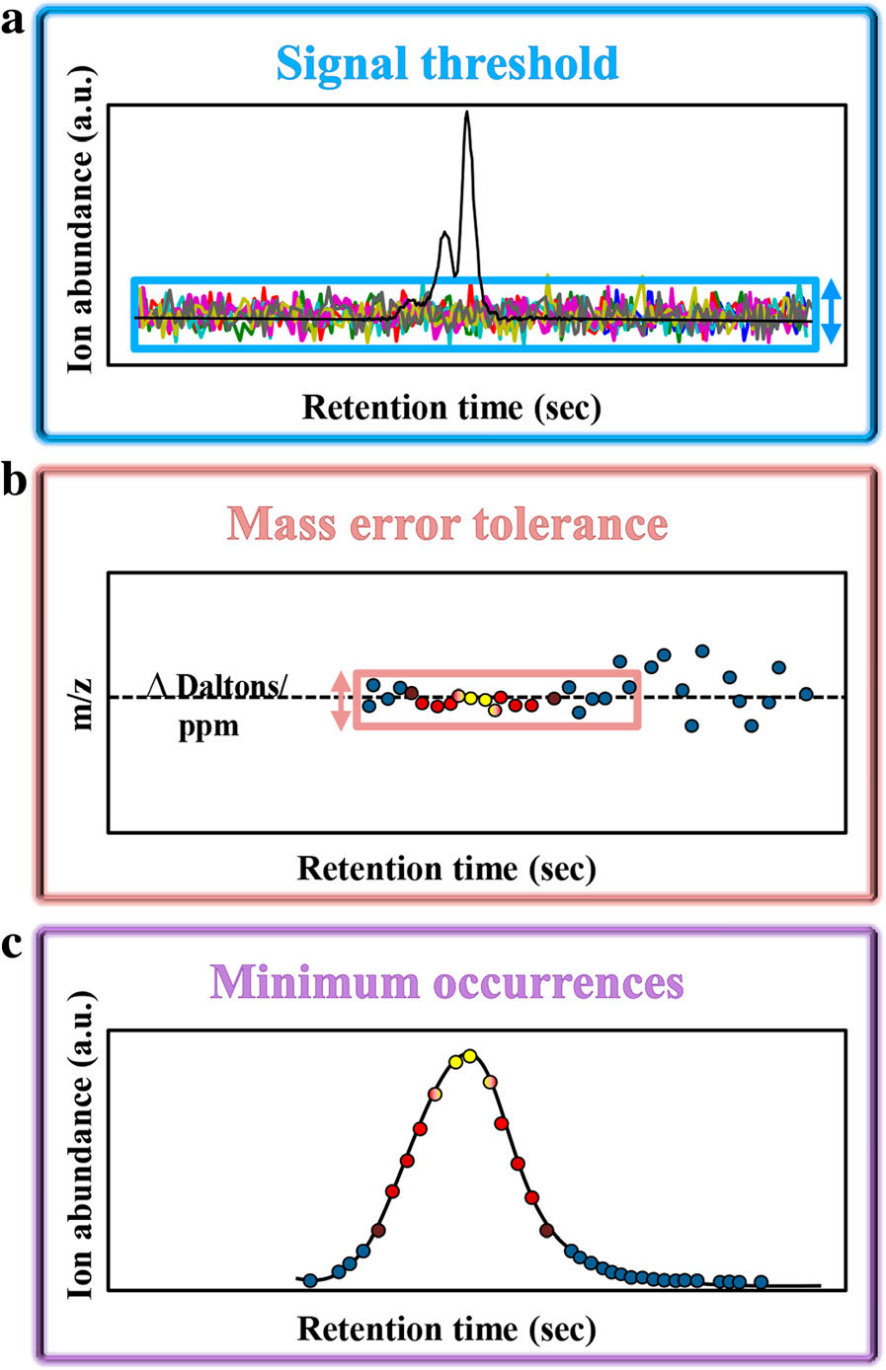
Parameters necessary to define an ROI. **a** Signal threshold, **b** Mass error tolerance and **c** Minimum occurrences

The aim of the ROI searching procedure is to scan for regions containing interesting mass traces, i.e., regions that include data at a relevant MS intensity (greater than a threshold value, Fig. 2a), enclosed within a specific mass accuracy or mass error tolerance (Fig. 2b) and constituted of a minimum number of occurrences (Fig. 2c).

**Fig. 3:**
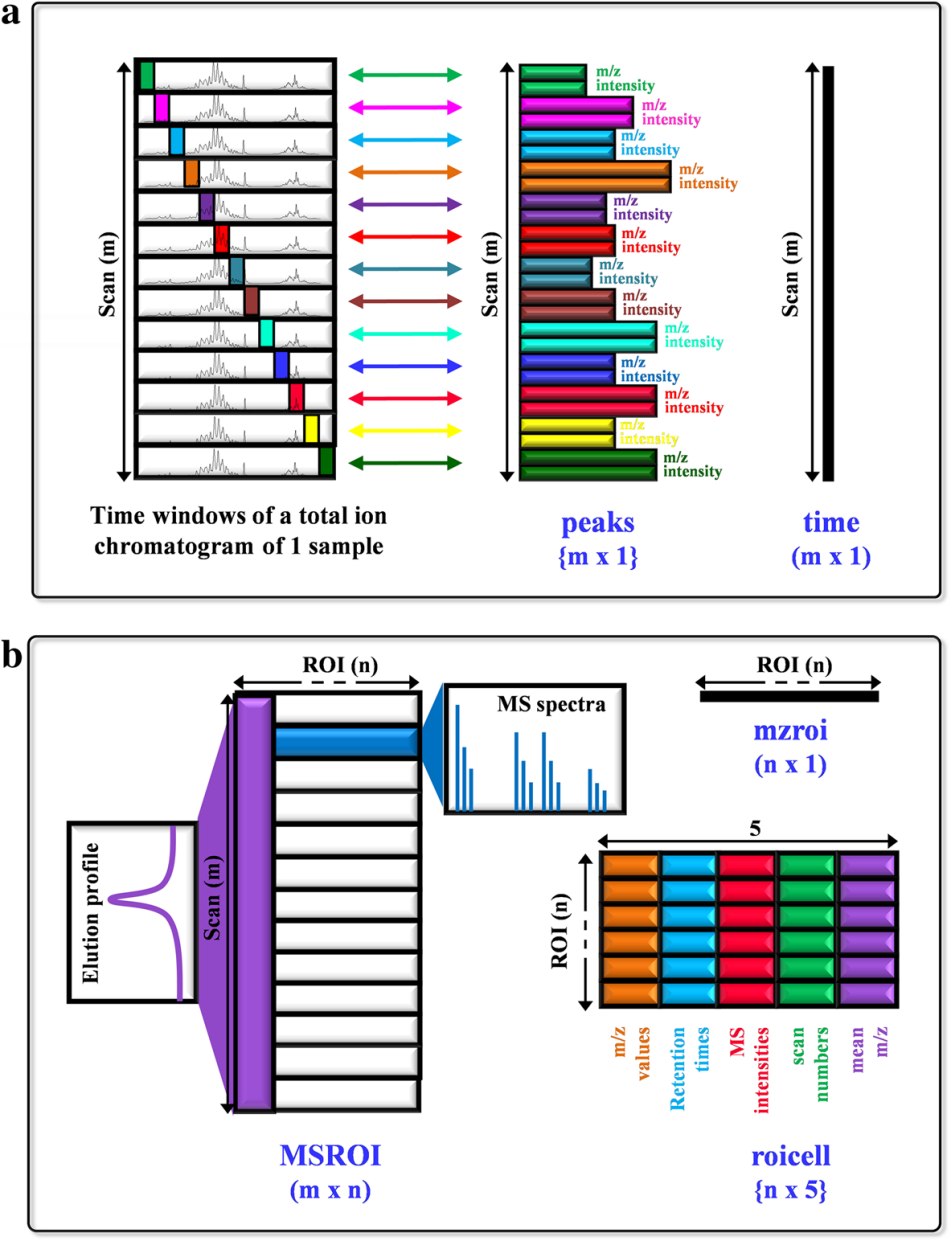
Schematic illustration of input (**a**) and output variables (**b**) of an ROI searching, filtering and compression algorithm. Data of the LC-MS chromatogram is described as a {m × 1} cell array (named as peaks), with m cells (equal to the number of retention times), each of them containing two vectors (of variable length among cells), corresponding to the m/z and intensity values acquired by the instrument at each of the retention times. Peaks and vector time (m × 1) are the input variables of ROI function together with the parameters required to define one ROI (thresh = 750, mzerror = 0.05 and minroi = 10 are used in this example), resulting in a data matrix, a data vector and a cell array (MSROI, mzroi and roicell, respectively) after ROI search. ROI (n) is the total number of ROIs obtained (in the example of the figure, nROI = 297). MSROI is a (m x ROI (n)) matrix, containing the MS spectra of every retention time in its rows, and the chromatograms of every ROI in its columns, mzroi is a vector containing mean m/z values of ROIs and roicell is a {ROI (n) × 5} cell array, containing ROI (n) × 5 cells (in the example of the figure it would be 297 × 5 = 1485). Cells comprised in roicell variable from column 1 to column 4 contain single vectors in their structures (containing information of m/z, retention times, intensities and scan number of the data enclosed in the same ROI, respectively) whereas cells comprised in the fifth column (roicell {ROI (n),5}) contain single values (corresponding to mean m/z values of ROI)

These three parameters are the input variables required for one ROI search, together with a vector listing the retention times at which the instrument records the measurements (variable “**time**” in Fig. 3a) and a cell array (i.e., array containing data of varying types and sizes in the MATLAB environment) containing the m/z values and MS intensities at each retention time (variable “**peaks**” in Fig. 3a). Interestingly, the m/z values (and their corresponding MS intensities) measured by the mass spectrometer at each retention time do not follow a regular pattern (i.e., the m/z measurements are not equidistant and may differ among mass spectra) and, therefore, the generated vectors enclosed in the cell array containing this information have distinct lengths. Figure 3a shows a representation of the pairs of vectors (i.e., one vector of the pair containing m/z values and the other containing MS intensities) including information from one LC-MS sample. Notably, the length of these vectors varies at distinct retention times, indicating that the mass spectrometer acquires distinct m/z values during each scan.

Once the input parameters are introduced, the ROI algorithm performs the ROI search using the following steps:

1. Search for m/z values associated with MS intensities greater than a signal threshold value (e.g. 0.1–1% of the mean/maximum signal intensity) in the first scan.
2. Search for clusters of m/z values enclosed within a specific mass error tolerance in the same scan.
3. Calculate the mean mass (or alternatively the median mass) of all the m/z values classified inside the same cluster (mzroi).
4. Arrange mean mass values from the lowest to highest values.
5. Repeat steps 1–4 for the remaining scans, merge them within the mass error tolerance and update the calculated mean mass values.
6. Select clusters having a minimum number of occurrences of m/z values.
7. Eliminate empty spaces in the final MSROI matrix, substituting them for random values with a mean threshold value, such as 1% of the threshold intensity value used in step 1.

The ROI search yields three outputs. A vector containing final mean m/z values of ROIs (“**mzroi**” in Fig. 3b), a newly arranged data matrix containing the MS spectra of every scan in its rows and the chromatograms of every ROI in its columns (“**MSROI**” in Fig. 3b) and a cell array (“**roicell**” in Fig. 3b) containing information about the m/z values, retention times, MS intensities, scan numbers and the calculated mean/median m/z value for each ROI.

### ROI search in more than one LC-MS sample

Since the main purpose of metabolomics is to study the differences in metabolic profiles between multiple samples (e.g., controls vs. exposed), the final data analysis must consider all samples simultaneously. In fact, an MCR-ALS analysis of multiple samples requires the construction of column-wise augmented data matrices (see Simultaneous MCR-ALS analysis of multiple samples section). The construction of these matrices is only possible when dimensions in the m/z mode of all individual data matrices are the same. However, data compression using the ROI strategy produces data matrices with m/z mode dimensions equal to the number of ROIs, which can vary between samples. Thus, a final unification of ROIs among samples, considering both common and uncommon mzroi values, must be performed.

The following description of the ROI search among multiple samples allows the construction of column-wise augmented data matrices that are suitable for a subsequent MCR-ALS analysis (Simultaneous MCR-ALS analysis of multiple samples section). The search for ROIs in several data files (LC-MS samples) is based on the determination of their common and uncommon ROI values. The ROI searching procedure among samples and the corresponding matrix augmentation procedure are performed successively between two MSROI data matrices, i.e., between two individual matrices, between one individual matrix and one augmented matrix or between two augmented MSROI matrices. Different strategies can be designed depending on the case. For instance, when ROI searching and matrix augmentation are performed first for control samples and then treated samples separately, the matrices can be further augmented together. The different steps of the algorithm for ROI searching and augmentation are presented below.

1. Check mzroi values between the two data matrices within the mass error tolerance, +/− mzerror. Consider the new mzroi to be the average of these values.
2. Build the new column-wise augmented data matrix with MS intensity values of the coincident mzroi values (if more than one mzroi value is coincident, then consider the sum of the MS intensity values).
3. Examine non-matching mzroi values; these values are accepted if their MS intensity is greater than the preselected threshold value. For the non-coincident mzroi values, replace empty values with random values at a low percentage (e.g., 1%) of the threshold intensity value.
4. Eliminate those mzroi values that are not coincident with an MS intensity value less than the threshold.
5. Reorganize the columns of the new augmented data matrix according to the new mzroi values, from lower to higher mzroi values.
6. Store output variables and plot ROI augmented matrices.

Thus, the required input information to perform ROI augmentation consists of the arrays of samples to be augmented, including m/z values (mzroi matrices) and MS intensities (MSROI matrices), the admissible mass deviation, the threshold intensity value and the vector containing the retention times. The output variables consist of a vector containing final mean m/z values of common and uncommon ROIs, the final augmented ROI matrix containing compressed data of all the input files and a vector containing the total number of scans (i.e., sum of the number of retention times of individual samples).

### Multivariate curve resolution-alternating least squares (MCR-ALS)

**Fig. 4:**
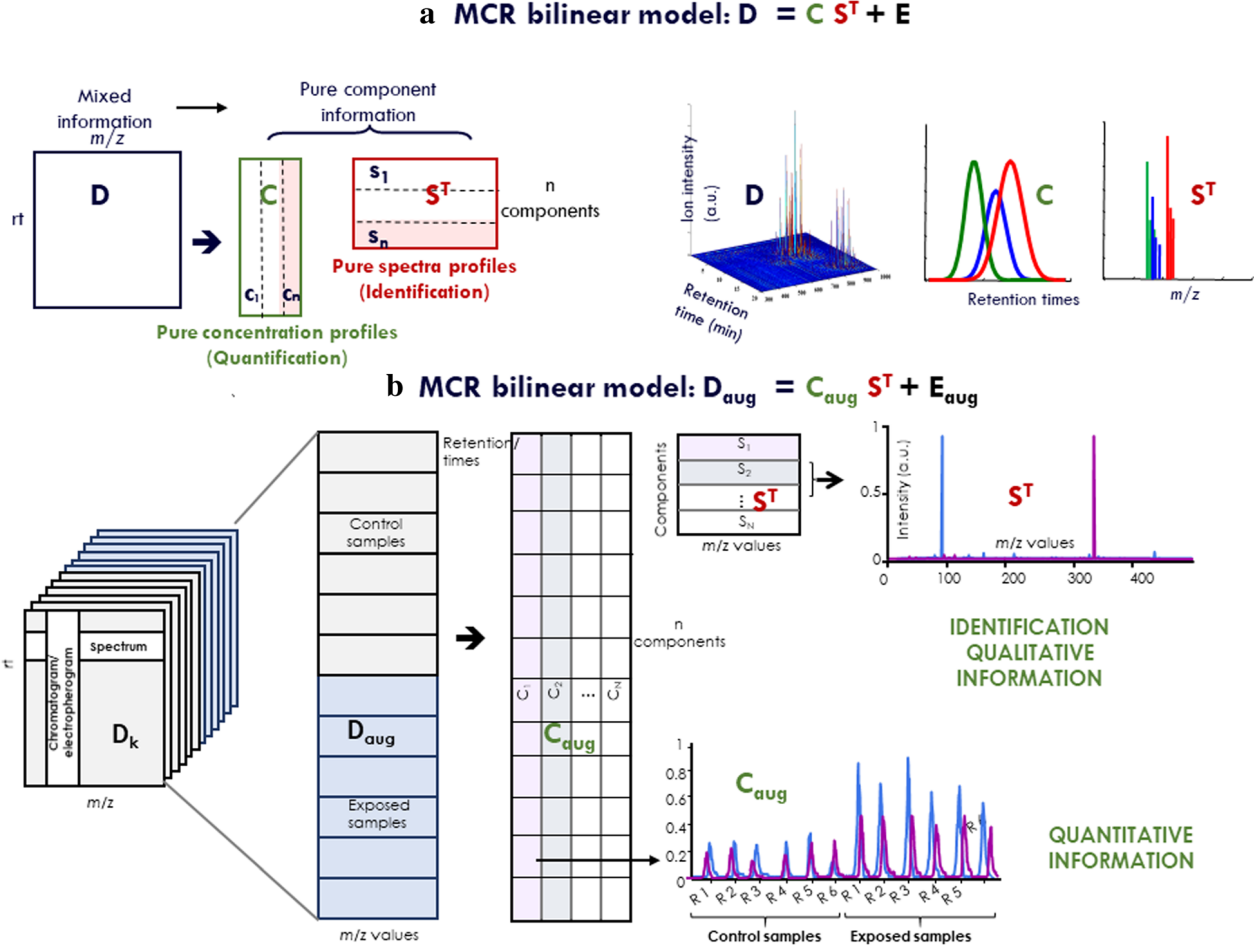
Graphical representation of the MCR bilinear factor decomposition model. **a** MCR bilinear model of the data matrix, D, obtained in the LC-MS analysis of one single sample. **C** and **S**^**T**^ are the factor matrices which have respectively the concentration (elution) and mass spectra profiles of the MCR resolved components in the analysed sample. **b** MCR model of the column-wise augmented data matrix, **D**_**aug**_**,** obtained in the simultaneous analysis of multiple individual, **D**_**k**_, data matrices, **C**_**aug**_ and **S**^**T**^ are the factor matrices which have respectively the concentration (elution) profiles of the MCR resolved components in each of the multiple simultaneously analysed samples and the common mass spectra profiles on all of them

The MCR-ALS method performs a bilinear decomposition of individual datasets, according to Eq. (1). In Fig. 4a, this bilinear model is graphically explained for the analysis of a single LC-MS sample/dataset.

1 $$ \mathbf{D}={\mathbf{CS}}^{\mathbf{T}}+\mathbf{E} $$

In this equation, matrix **D** (I x J) exemplifies the spectral dataset derived from the output of a mass spectrometer. For LC-MS data, matrix **D** includes the MS spectra measured at all chromatographic retention times (i = 1, … I) in its rows and the elution profiles at the complete range of spectra m/z channels (j = 1, … J) in its columns. This matrix is decomposed in the product of two small factor matrices, **C** and **S**^**T**^. The **C** (I x N) matrix encloses column vectors that agree with the concentration elution profiles of the N (*n* = 1, …, N) pure chemical constituents or components of matrix **D**. In the **S**^**T**^ (N x J) matrix, row vectors correspond to the MS spectra of these N pure components. The fraction of **D** that is not described by the bilinear model constitutes the residual matrix **E** (I x J). MCR-ALS methods presume that the measured variance in all samples in the raw dataset is explained using a combination of a relatively small number of chemically significant profiles compared to the number of measured variables (in this case, the number of ROIs). For LC-MS datasets, the variance observed in the investigated data matrices is explained by the combination of a number of components defined by their pure mass spectra (row profiles in the **S**^**T**^ matrix) weighted by their concentration profiles (elution profiles in **C** matrix), as given in Eq. (1). Every component resolved by MCR-ALS is characterized by its unique MS spectrum and its elution profile, and are interpreted directly. The **C** and **S**^**T**^ solutions of Eq. (1) are obtained using an alternating least squares (ALS) optimization under preselected constraints [1, 3, 22–25]. In the case of LC-MS data, due to the sparsity of the MS data, non-negativity constraints of the elution and mass spectra profiles of the resolved components already provide good solutions for **C** and **S**^**T**^, although other constraints may be applied to the profiles of the resolved components, such as unimodality and local rank or selectivity constraints [3]. The MCR-ALS method has been described in previous studies and applied to different type of datasets [1, 3, 22–25].

The number of metabolites/lipids that is ultimately resolved by the proposed procedure will depend on different experimental parameters, such as the efficiency of metabolite extraction, the suitability of the chromatographic column, the resolution power, signal to noise ratio of the mass spectrometer, and the size of the elution time window analyzed. The number of selected components in the ROIMCR procedure, N, should be sufficiently large to capture all data features related to metabolites. Unavoidably, in addition to the metabolites, other MS signal contributions (background, solvent, etc.) are simultaneously resolved and yield extra components. Therefore, the recommendation is to select a number of components that is sufficiently large to explain most of the variance in the experimental data. The total number of components resolved using MCR-ALS is limited by the intrinsic mathematical structure of the dataset analyzed. MCR-ALS uses linear algebra operations to solve (using a least squares method) the system of linear equations involved in the assumed bilinear model (Eq. (1)) used to analyze the experimental data. The solution of this model implies the inversion of matrices **C** and **S**^**T**^, and therefore implies that their columns and rows, respectively, are linearly independent. This solution is also related to the rank of the experimental data matrix **D**. Different datasets will enable the resolution of a different number of components. If the number of components proposed is too large, the inversion of **C** and **S**^**T**^ matrices is not possible due to rank deficiency problems. Occasionally, the precise definition of the best number of components is difficult to obtain due to the experimental noise; nevertheless, those extra components that are only related to noise will provide the shapes of the elution and spectra profiles that are unfeasible from a chemical perspective and explain very low data variance. No additional components should be added without a significant increase in the explained data variance, and should have well-shaped single peak elution profiles and sparse MS spectra signals. Once the results are obtained, every resolved component is examined to confirm its reliability and for its identification (MS) and relative quantitation (elution profiles). This output examination is performed individually, component by component. Residuals are also examined to determine whether some well-shaped peak chromatographic signals are still present. In some cases, some minor components with a very low contribution that is very close to the noise level are unable to be distinguished from background noise in the residuals. This situation is a possible limitation of untargeted metabolic approaches. However, most of the untargeted metabolomic studies focus on changes in the concentrations of the metabolites caused by the investigated stress conditions, not their absolute concentrations. Another possible alternative, in some cases, is to subdivide the whole chromatographic run into different time windows and submit each of them to a deeper MCR-ALS analysis, where the presence of minor components is analyzed more extensively.

### Simultaneous MCR-ALS analysis of multiple samples

MCR-ALS has been simultaneously applied to distinct datasets or matrices. For instance, the simultaneous analysis of multiple samples using LC-MS is accomplished by generating column-wise data matrices (**D**_**aug**_) including different data matrices related to distinct chromatographic runs appended one above the other. Therefore, the MS spectral (column) direction is the same for all matrices and the data matrix extent is augmented in a column-wise manner in the chromatographic (rows) direction. The bilinear model decomposition of the column-wise augmented matrices, **D**_**aug**_, in the analysis of multiple LC-MS samples (data sets) is presented in Eq. (2) and displayed graphically in Fig. 4b.

2 $$ {\mathbf{D}}_{\mathbf{aug}}={\mathbf{C}}_{\mathbf{aug}}\ {\mathbf{S}}^{\mathbf{T}}+{\mathbf{E}}_{\mathbf{aug}} $$

In this case, resolved pure mass spectra are the same for all simultaneously analyzed chromatographic runs or experiments (**S**^**T**^), while elution profiles (**C**_**aug**_) can vary from run to run.

In the MCR-ALS method, bilinear models described in Eq. (1) (single data matrix illustration) or Eq. (2) (augmented data matrix illustration) are resolved using an alternating least squares optimization approach under constraints [3]. In both cases, when considering metabolomic LC-MS data, the minimum constrains to apply consist of non-negativity for concentration (elution), **C** or **C**_**aug**_, and spectra, **S**^**T**^, profiles, and normalization for the second. Due to the sparse nature of the MCR-resolved elution profiles, particularly the MS spectra profiles, no additional constraints are required to achieve reliable results.

In the proposed ROIMCR procedure, individual or augmented MSROI data matrices (**D** or **D**_**aug**_) are submitted for MCR-ALS analysis. The application of this method will provide the concentration/elution, **C** (or **C**_**aug**_), and MS spectra, **S**^**T**^, profiles of the resolved components. Notably, in the MCR-ALS procedure, elution profiles in **C**_**aug**_ are not required to be aligned or shape modelled among different samples (chromatographic runs), and spectra profiles are the filtered MSROI-compressed spectra with the full instrument mass accuracy. Peak areas are calculated by integrating (numerical summation) the values in the concentration (elution) profiles resolved using MCR-ALS. These profiles are located in the columns of the **C** matrix (Eq. (2)) for every simultaneously analyzed sample. The summation is performed computationally. Depending on the time acquisition of the LC-MS instrument, the peak profile will be digitized with a different number of values, which would usually imply a minimum of 5 intensity values, and in many circumstances, this profile contains more than 10 intensity values. If the concentration profile does not have a peak shape, it is discarded and not considered. Most, but not all, of the elution profiles resolved using MCR-ALS have a good peak shape. For instance, background, solvent, and other spurious signals do not display a good peak shape and are not further considered. The number of components in the analysis of the **D**_**aug**_ matrix (simultaneous analysis of multiple samples or datasets) is selected in a similar manner as described above for the analysis of a single dataset, after considering the increased complexity of the augmented data matrix **D**_**aug**_ compared to the individual **D**_**k**_ matrices (see Fig. 4). Again, a more detailed description of the MCR-ALS method and the implementation of different constraints is presented in previous publications [1, 3, 22–25].

## Datasets

The dataset used to illustrate the performance of the current methodology was obtained from a previous study performed by the authors [16, 17], where LC-MS data for lipids extracted from human placental choriocarcinoma cells (JEG-3) that were exposed to DMSO (vehicle controls) and to a non-lethal dose of the chemical endocrine disruptor TBT (exposed samples) for 24 h. Both groups (i.e., controls and exposed) contain three replicates. These raw data sets are available in CDF format at http://cidtransfer.cid.csic.es/descarga.php?enlace1=5792320ab8143eca122f4cf7dbb68cd40e2cf7.

Thus, the interested reader can use the data to test the ROIMCR procedure presented here. For details regarding the characteristics of the data, readers are advised to consult: https://www.nature.com/protocolexchange/protocols/4347.

Results of the application of the ROIMCR procedure to other datasets from recent studies [16–22, 26–28] are briefly described in “Applications of the ROIMCR procedure” section.

## Implementation of the ROIMCR procedure

The ROI compression procedure presented in this study has been implemented as command line functions in the MATLAB environment available at http://cidtransfer.cid.csic.es/descarga.php?enlace1=298348e5b34daf9e844835352bafa645250ee1 and at www.mcrals.info.

A new user-friendly graphical interface for ROI compression is currently being developed and will be freely available at the same site. The provided MATLAB functions for ROI searching, filtering and compression are related to: a) ROI searching in one sample (ROIpeaks function); b) the evaluation of ROI profiles (ROIplot function), and c) the generation of augmented ROI data matrices (MSROIaug function). In addition, a statistical evaluation of the concentration profiles obtained after the MCR-ALS analysis may be performed (plot_profiles_table function). Regarding the implementation of MCR-ALS, its user graphical interface is also available at www.mcrals.info.

## Results

Although the dataset used as example in the present study was already used in previous studies by the authors [16, 17], the results presented here were not presented in the previous publications and are specifically selected to show the key features of ROIMCR methodology in the present study. These results include ROI searching of individual datasets, ROI data matrix augmentation and MCR-ALS analysis of the obtained augmented ROI matrix. The readers interested in the LC-MS data conversion and MATLAB import procedure are advised to consult https://www.nature.com/protocolexchange/protocols/4347.

### ROI searching procedure

#### Optimization of ROI parameters

As previously stated in the Methods section, some parameters must be optimized prior to the search for ROIs. The example presented in Table 1 shows the results of the ROI search after setting distinct values for one of the three input parameters, while maintaining the values for the other two parameters unchanged. In all cases, three distinct values are tested for the parameter: 10 times higher than the recommended value, the recommended value, and 10 times lower than the suggested value. In the first case, where the influence of the threshold on ROI search was evaluated, the three options tested corresponded to threshold values of 7500, 750 and 75 a.u. (a search using ppm values instead of a.u. is also considered). The recommended threshold value should be adjusted between 0.1–1% of the maximum measured MS intensity. Since the maximum measured MS intensity of the evaluated sample was 3.5118·10^5^ a.u., the recommended threshold value would be between 351.18 and 3511.8 a.u. In particular, we selected an intermediate value of 750 a.u. as the optimum value. The higher and the lower values tested (7500 and 75 a.u., respectively) were chosen to clearly show that a decrease in the threshold value produces an increasing number of ROI values, together with a substantial increase in the computation time (see Table 1, in seconds), while an increase in the threshold value results in the opposite changes. Hence, the threshold value must be adjusted with caution since it can increase data quality by eliminating noise, but immoderate threshold values may result in information loss. In fact, this parameter is better visually evaluated from the graphical outputs to ensure that it results in noise diminution without signal loss or deformation.

**Table 1:** Number of ROIs and computation time resulting from ROI searches performed with three different values of the input parameters (signal threshold in absolute units, a.u., mass error tolerance in Da/e, and minimum number of occurrences). In cursive are indicated the optimum values of the parameters. The results shown are obtained considering the variation of one parameter while the other two remain fixed in their optimum value

Parameters of the ROI search		Number of ROI	Computational time^a^ (s)
Signal threshold (a.u.)	7500	55	0.8
	*750*	*300*	*1.8*
	75	1357	8.8
Mass error tolerance (Da/e)	0.5	267	1.8
	*0.05*	*300*	*2.0*
	0.005	356	2.0
Minimum occurrences	100	23	1.7
	*10*	*300*	*1.9*
	1	449	1.9

^a^ Computational time using a 64-bit Windows Intel(R) Core™ i5–3470 CPU computer of 8GB and version 8.2.0 (R2013b) of MATLAB

In the second case (see Table 1), the study of the effect of an admissible mass deviation on an ROI search, the three options tested corresponded to mzerror values of 0.5, 0.05 and 0.005 Da/e. The optimum mass deviation value should be halfway between an excessive and an insufficient mass accuracy. In this example case, with an mzerror value of 0.005 Da/e, peaks corresponding to the same ion were divided into distinct parts, whereas for a value greater than 0.5 Da/e, the opposite situation occurred, and peaks corresponding to distinct ions collapsed into the same chromatographic signal. Thus, the optimum mzerror value was set to 0.05 Da/e. The higher and lower values tested (0.5 and 0.005 Da/e, respectively) were again selected to easily visualize their effects on final ROI selection. Similar to the threshold parameter, a decrease in mzerror value increased the number of ROIs. In this case, however, the increase in ROI number was not as spectacular as for the threshold parameter, and the elapsed computation time was fairly constant for all calculations (see Table 1). In the third case (see Table 1), an evaluation of the effect of minimum occurrences on an ROI search, the three values tested corresponded to 100, 10 and 1. The minimum number of occurrences is directly related to a range of peak widths and detector speed, which varies among high-performance liquid chromatography (HPLC) (20–50 s) and ultra-high-performance liquid chromatography (UHPLC) (5–12 s) systems. In the current representative case, the system used to analyze the sample was an Acquity UHPLC system, and thus the optimum number of occurrences should correspond to a peak with range of 5–12 s. In particular, with this instrumentation, the interval between each occurrence was 0.63 s, and thus we selected 10 occurrences (i.e., 6.3 s) as the optimum value. When considering results obtained for the three values tested, the same trend observed for the other parameters was again detected, as higher numbers of ROIs were obtained when the values of the minimum number of occurrences decreased and lower numbers of ROIs were observed when the value increased. Regarding the mzerror parameter, the increase in ROI number observed at a lower minimum number of occurrences was less substantial than for the threshold parameter, and the elapsed computational time was similar in the three calculations (see Table 1). The example presented here clearly illustrates the importance of the proper optimization of ROI parameters before the application of the method. It also highlights the influence of the particular instrumental specifications (e.g., mass accuracy) on these parameters.

#### Evaluation of ROI profiles

**Fig. 5:**
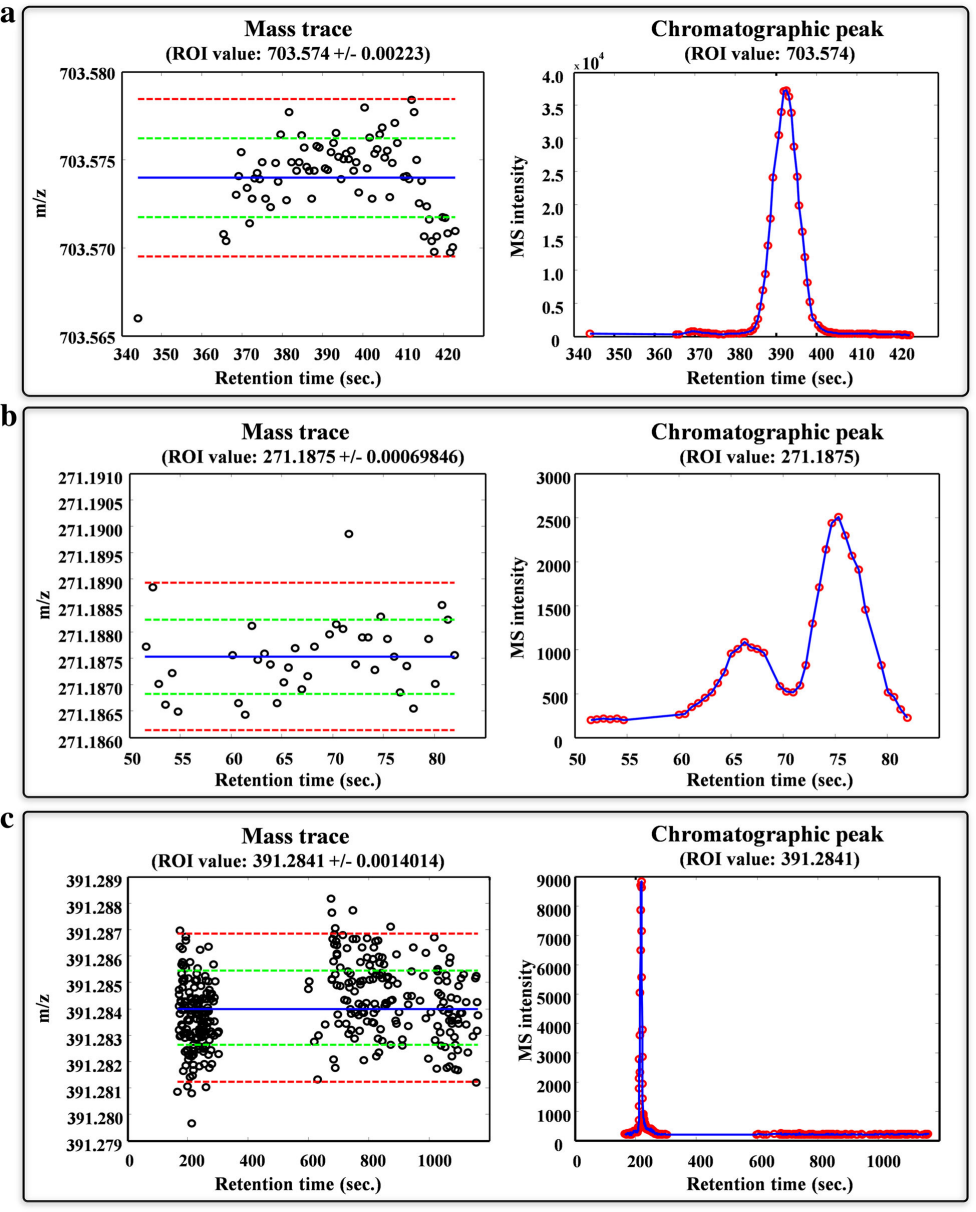
Representation of the chromatographic elution profiles and mass traces of three ROI values: **a** one ROI with a single-peak elution profile, **b** one ROI with a double-peak elution profile and **c** one ROI with an elution profile with two clearly distinguished regions, corresponding to a defined peak and the baseline/background noise

After the ROI search in individual matrices, their profiles were evaluated to determine whether they fit the chromatographic shape of the original data. Figure 5 shows the two distinct graphical representations of three ROIs obtained from the Control 1 sample after the ROI searching, filtering and compression steps. The three selected ROI correspond to the m/z values of 703.5740 Da/e (Fig. 5a), 271.1875 Da/e (Fig. 5b) and 391.2841 Da/e (Fig. 5c). The selected ROIs exhibit three completely distinct elution profiles and related mass distributions. In the first case (Fig. 5a), the elution profile of the ROI with an m/z of 703.5740 Da/e describes a single-peak curve and the corresponding mass distribution is appreciably regular over time. The second case (Fig. 5b) corresponding to an ROI with an m/z of 271.1875 Da/e is particularly interesting since it describes a double-peak curve. As observed in the mass spectrum for this ROI, three slightly distinguishable regions of mass measurements are presented, corresponding to the initial measurements of the profile curve, first peak and second peak. This ROI may correspond to different isomeric chemical compounds resolved by the chromatographic column that have equal m/z values at the considered mass deviation. Finally, in the third case (Fig. 5c), the elution profile of an ROI with an m/z of 391.2841 Da/e distinguishes two clusters of MS points. The first cluster, located at approximately 200 s, is associated with the chromatographic peak, whereas the second cluster, located between 600 and 1200 s, is related to the background noise. The representations of mass traces provide valuable information about the nature of experimental MS measurements. In general, this information is unknown to MS users and may be crucial for a better analysis and optimal interpretation of LC-MS data.

Once the optimum parameters for the ROI search were selected, the augmentation was performed and a final augmented ROI matrix was generated. The dimensions of that matrix were (11,394 × 481), the x-dimension corresponding to six times the number of retention times of one sample (i.e., 1899) and the y-dimension corresponding to the total number of common and uncommon ROIs among the six samples.

#### ROI profiles versus feature profiles of XCMS

Various forms (X) of chromatography and mass spectrometry (XCMS) is a popular data analysis software package used by the metabolomics community that enables the automatic processing of large size full-scan LC-MS datasets and the prediction of candidate metabolites using mass identification and retention time algorithms [29]. It is based on feature detection, where a “feature” is defined as a single m/z measurement of the mass spectrometer. A general XCMS analysis starts with the application of the centWave data processing algorithm, which first identifies features using the ROI approach and then models the obtained chromatographic peaks using a wavelet transformation and a Gaussian shape curve fitting strategy. In the last step, some alignment algorithms (such as obiwarp) are used to align the chromatographic peaks of the same feature among distinct samples.

Due to the resemblance to the ROI search performed in the first step of the centWave algorithm of XCMS software and the search performed using our methodology, the results obtained using both approaches were compared in the present study. We used the virtual research environment built on the Galaxy web-based platform technology called Workflow4Metabolomics (W4M) [10, 30] www.msomics.com/ version 3.0, which incorporates XCMS and CAMERA packages. When using the XCMS package included in W4M, the first step requires the users to define the MS parameters prior to the ROI search. These parameters are similar to those defined in our ROI methodology and include: i) the m/z maximal error tolerance in consecutive scans (in XCMS software reported in ppm), ii) the minimum and maximum chromatographic peak width (reported in seconds) and iii) the signal-to-noise ratio threshold. Importantly, the optimization of the latter parameter (i.e., MS intensity threshold) is linked to three input arguments, which the users are asked to define. The first and second input arguments are used to perform a preliminary threshold filtration and are called the “prefilter intensity” and the “noise filter” arguments. Both are used as prefilter steps for the first selection phase to retain mass traces that contain peaks (i.e., prefilter peaks) with an intensity greater than the desired threshold (i.e., prefilter intensity). In the second selection phase, the third parameter that the user must define is the “signal/noise threshold”. Table 2 shows the number of ROIs obtained for all six samples investigated in this study (i.e., three control replicates and three tumor cell sample replicates treated with TBT) using the two approaches, together with the input parameters utilized. The resulting number of ROIs is 481 for our ROIMCR methodology and 300 for the XCMS package (in the W4W platform). The difference may be attributed to the meanings of the input parameters used for their calculation with the distinct approaches that are similar, but not exactly the same, and therefore the input parameters are not completely comparable, particularly the threshold parameter. However, when observing the high-resolution m/z values associated with these ROIs, 251 of the ROIs (i.e., greater than 80% of the number of ROIs identified using W4W) coincide between the two strategies (within an m/z error of 15 ppm, see Additional file 1: Table S1), indicating that both approaches produce very similar results. These coincident ROIs have a greater MS intensity.

**Table 2:** Comparison of ROI search results obtained using our MATLAB routines and the centWave algorithm of XCMS package of Work4Metabolomics

	Number of ROIs	*m/z* error tolerance	Peak width	Threshold	
ROI search using MATLAB home-made routines	481	0.05 *Da/e*	10 s	750 a.u.	
ROI search using *centWave* algorithm of XCMS of W4W^a^	300	5 ppm	10 s	S/N threshold	10
				*Prefilter intensity*	7500
				*Noise filter*	0
Number of coincident ROIs	251				

^a^ The version 3.0 of Work4Metabolomics available at http://workflow4metabolomics.org/ has been used to do the calculations

In addition to the comparison performed here, other recent studies comparing the performance of XCMS software to an ROI search followed by MCR resolution are presented in the literature. Recently, the proposed procedure was tested in different studies, where the complexity of the analyzed samples was considerably greater and the number of samples larger (see Navarro-Reig et al. [26] and other citations listed above [16–21]. We have also validated the procedure for quantitative purposes in Dalmau et al. [27] All these results have confirmed the adequacy of the proposed ROIMCR strategy to analyze metabolomic data, leading to very similar conclusions in both cases (XCMS and ROIMCR).

These advantages are briefly described below.

- A peak alignment strategy is not required (needed in XCMS).
- The shape of chromatographic peaks/elution profiles does not need to be modeled (needed in XCMS).
- All features in the mass spectrum of one metabolite/lipid are directly resolved in the same MCR component. The assignment of different features to the same component spectrum is unnecessary (needed in XCMS). The CAMERA procedure is not required (needed in XCMS).

Additionally, other advantages of XCMS are also present in the ROIMCR procedure.

- The full mass accuracy of MS measurements is preserved (the ROI searching procedure is similar to the one used in XCMS).
- Signal filtering and data compression properties are also derived from the ROIMCR procedure.
- The open source code is available (see the links below).

### Data resolution using the MCR-ALS analysis

Once the augmented data matrix of ROI compressed data from the six samples has been constructed, the next required step is the MCR-ALS analysis.

The selection of the number of pure components is the first step in the MCR-ALS analysis. As described in “Multivariate curve resolution-alternating least squares (MCR-ALS)” section, the optimum number of MCR-ALS components should be sufficiently large to explain all the chromatographic peaks, the background (e.g., solvent), and contributions from other unknown signals. Any increase in the number of components should produce a significant reduction in the lack of fit and a corresponding increase in the explained variance. Otherwise, no other components should be added to the calculation. In the example presented here, the number of components was proposed to be 50 for the MCR-ALS analysis of the augmented matrix, resulting in a less than 7% lack of fit and 96.5% of the variance was explained. A larger number of components did not significantly improve the lack of fit or model new chromatographic peaks. The difference between the larger number of ROIs (481) compared to the smaller number of MCR components resolved (50) has two potential explanations. The first explanation is that not all ROIs will produce different MCR components within an elution profile and a mass spectrum profile characteristic of a metabolite or lipid. In addition, another important explanation is in MCR-ALS, various ROI (features) are grouped into the same component. ROIs grouped into the same MCR-ALS component generally include isotope and adduct peaks. In fact, the capability of MCR-ALS to group features corresponding to the same component (metabolite/lipid) is one of the most distinguishing and advantageous aspects of our ROIMCR methodology compared to other tools such as XCMS that associate each feature with a unique m/z. For this reason, another package, CAMERA [7, 31], has been developed to search for features that correspond to the same compound. In the present study, we used the CAMERA package of W4W to search for ROIs obtained with the centWave algorithm that corresponded to the same compound. The results of the CAMERA search indicated that the initial 300 ROIs were grouped into 194 components. However, the larger number of components obtained using the CAMERA software than the number of components resolved with MCR might be attributed to the fact that not all the 194 components correspond to distinct chromatographic peaks and further grouping should be performed, which is a laborious task. The final list of lipids or metabolites obtained using the two methods should ultimately be comparable, which implies their identification based on their exact mass or another analytical strategy (see Biomarker discovery section).

Importantly, due to the sparse number of MS spectra and their high selectivity, their resolution has little ambiguity [32, 33] and the possible underestimation of the number of MCR-ALS components will not cause a misinterpretation of the results but only a small loss of information. In that case, the final interpretation will only be provided for the ultimately resolved components. As previously explained in “Multivariate curve resolution-alternating least squares (MCR-ALS)” section, another possibility to resolve metabolites with very low signal contributions in LC-MS untargeted studies is to divide the whole dataset into shorter elution time windows.

### Biomarker discovery

Concentrations and spectral profiles of the resolved MCR-ALS components are finally used for biomarker assessments. However, a subsequent statistical analysis is required to identify the most relevant MCR-ALS components (i.e., the components that significantly vary among control and stressed samples). Distinct statistical tests have been used for this evaluation, such as the classical Student’s t-test, which was used in the present study. This test, together with other statistical tests, may be performed using the functions and protocol [23] available at https://www.nature.com/protocolexchange/protocols/4347.

**Fig. 6:**
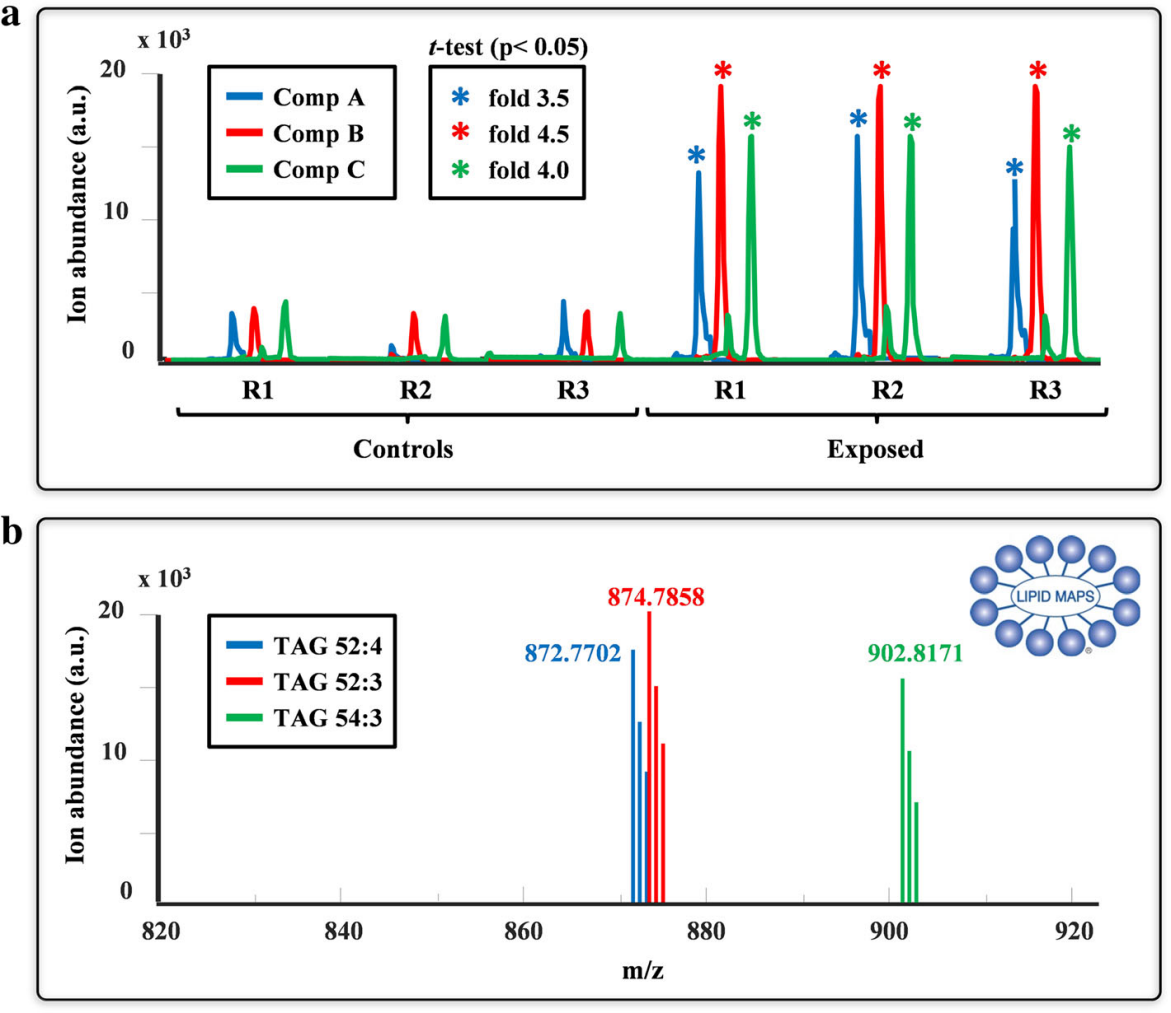
Elution profile (**a**) and mass spectra (**b**) of three resolved MCR-ALS components. * Statistical significant differences respect to the control (*p*-value < 0.05). Lipid Maps was the MS reference database used for the identification of lipids

Figure 6 shows a representation of the elution and spectra profiles of three representative MCR-ALS resolved components. As shown in the elution profiles of these components (Fig. 6a), a noticeable difference in the areas and heights of the chromatographic peaks is observed among control and exposed samples. This difference indicates an increase in the levels of these lipids after the treatment with TBT.

A classical statistical Student’s t-test was performed on each component using a *p*-value less than 0.05 as the criterion to evaluate the significance of these changes. The results of the test revealed significant changes in the heights of the three components between the two groups (i.e., controls and exposed), suggesting that they represent potential biomarkers for TBT exposure. When needed, multiple comparisons procedures (MCPs) [34] can be applied to avoid the assignation of false positives. These statistical procedures are intended to consider and suitably manage multiple effects through some shared or joint measure of mistaken inferences. Alternatively, ANOVA and its multivariate extensions for well-designed data have been applied [35, 36] to better ascertain the reliability of the observed effects of TBT exposure. Additionally, the fold-changes for the three components were calculated (Fig. 6a), resulting in 3.5-fold, 4.5-fold and 4.0-fold changes for components A, B and C, respectively. The MS spectra profiles were evaluated to identify the lipid species corresponding to these MCR-ALS components. As shown in Fig. 6b, the exact masses of components A, B and C were 872.7702, 874.7857 and 902.8171 Da/e, respectively. Further identification using MS databases such as Lipid Maps [37] is also possible. As shown in the same figure, components A, B and C corresponded to triacylglycerol species 52:4, 52:3 and 54:3, respectively. Notably, this identification was made possible to a large extent because no loss of mass spectral information occurred after ROI compression.

## Applications of the ROIMCR procedure

In previous sections of this study, the different methodological aspects of the ROIMCR procedure have been described in detail for a single dataset as an example. In previous and simultaneously performed recent studies [16–22, 26, 27], the ROIMCR procedure has been applied to diverse datasets and scenarios, such as a recent investigation of the rice metabolome using LCxLC-MS/MS [17]. In this study, the ROIMCR procedure was applied to the different modulations of the second LC column to analyze several samples arranged in a super column-wise augmented data matrix. The number of components resolved in the MCR-ALS analysis of this super augmented data matrix was 250. The ROIMCR method determined which of the mass traces belonged to every resolved metabolite, and the resolution of the sample metabolites in the entire dataset was much faster with this method than with other traditional strategies based on the analysis of each component separately. In another recent study [19], the effects of different endocrine disruptors (EDC) on zebrafish (*Danio rerio*) embryos were investigated using an untargeted LC-HRMS (Exactive Orbitrap) metabolomic analysis. In this case, 25 zebrafish embryos (5 replicates for each of the 5 applied chemical doses) were simultaneously analyzed using the ROIMCR method for every EDC treatment. Eighty-six to 110 MCR-ALS components were resolved, depending on the EDC used, and the corresponding changes in the metabolite concentrations suggested the presence of similar underlying zebrafish responses to the different investigated EDCs. The underlying metabolomic and lipidomic patterns linked to thermal acclimation in *Saccharomyces cerevisiae* were investigated in another study using a combination of H^1^NMR and LC-MS. In this example, the application of the ROIMCR procedure allowed for more than a 100-fold reduction in the computer storage requirement, but maintained the highest possible experimental mass accuracy. Twenty-four yeast samples cultured at different temperatures were simultaneously analyzed and produced 80 tentative lipid candidates in the ESI+ mode and another 50 lipids in the ESI- mode of MS. In another recent study [21], the proposed ROIMCR LC-MS approach facilitated an assessment of the effects of acute and chronic UV irradiation on the phenotype and lipidomic profiles of keratinocytes. Finally, a similar ROIMCR strategy was applied to the simultaneous analysis of multiple mass spectra from plants to investigate the changes in lipid composition induced by the application of the chlorpyrifos pesticide [18]. MS data from 20 samples receiving each treatment (4 doses with 5 replicates) at different growth stages were simultaneously analyzed and provided information about the changes in the spatial composition and distribution of different lipids on the surface of the investigated samples, which were also identified.

Finally, as an additional confirmation of the advantages of the ROIMCR procedure, the results obtained in the analysis of a new dataset are provided here to complete the assessment of this method. This dataset was obtained in a previous study [28] where three tissues (brain, gonads and gastrointestinal tract) were obtained from male and female zebrafish exposed to low dietary doses of four different carbon nanotubes (CBNs): C60 fullerene (C60), single-walled carbon nanotubes (SWCNT), short multi-walled carbon nanotubes (MWCNTs), and long multi-walled carbon nanotubes (MWCNTs). The lipid extracts of these samples were analyzed using LC-MS. The data produced in these LC-MS analysis were processed using the ROIMCR procedure (see Additional file 2: Figure S1, Additional file 3: Figure S2 and Additional file 4: Figure S3). One hundred fifty components were simultaneously resolved, explaining the 99.2% of the total data variance. Each of these components was described with one elution and one mass spectrum profile. Additional file 5: Figure S4 shows an example of the MCR-ALS-based resolution of component 2. In the upper panel of Additional file 5: Figure S4, an example of MCR-ALS output results is provided for the resolution of the 150 components in the simultaneous analysis of the same 80 zebrafish samples treated with the different carbon nanotubes. The lower panel Additional file 5: Figure S4 shows an example of the MCR-ALS-based resolution of component 2. This lipid component was identified from its mass spectrum as TAG 50:3, C_53_H_100_NO_6_, with an m/z value of 846.7474. Additional file 6: Figure S5 and Additional file 7: Figure S6 show the resolution of other MCR-ALS components, after their proper identification using lipid databases. In Additional file 6: Figure S5, the MCR-ALS components correspond to glycerolipid species, whereas in Additional file 7: Figure S6, they correspond to glycerophospholipid species. In all cases, the selected components are the most representative biomarkers of the treatments with the distinct carbon nanoparticles (i.e., C_60_, SWCNT, ShWCNT and LWCNT) in the distinct zebrafish tissues (i.e., brain, gonads and intestinal tracts). As observed in these figures, the differences in the numbers of MCR-ALS components between control and treated samples were very significant in some cases. For instance, in Additional file 6: Figure S5, concentrations of the TAG 54:3 and TAG 54:5 lipid species were up to 5-fold higher in the gonads of female controls compared to SWCNT-treated samples, as evaluated using MCR-ALS. In some other cases, however, a non-significant effect of the treatment was observed. An example is brain tissues from females presented in Additional file 7: Figure S6, which showed very similar LC-MS elution profiles for the resolved MCR-ALS components in controls and zebrafish treatment with the distinct carbon nanoparticles. More details and specifically a discussion of the results obtained in the study of this system are provided in a published study [28] and at 10.1093/mutage/gew050. Again, the valuable contribution of the MCR-ALS methodology to the evaluation of LC-MS omic profiles in target organisms exposed to environmental contaminants has been validated.

In summary, based on these previous studies, the applicability of the ROIMCR method has been confirmed for diverse metabolomic and lipidomic studies and presents some advantages compared to other strategies, as explained in “ROI searching procedure” and “Data resolution using the MCR-ALS analysis” sections of this paper. Moreover, XCMS and MCR-ALS data analysis strategies were also compared in other studies, such as in the previously published LC-MS metabolomics data analysis protocol [22], in the review article describing data analysis strategies for targeted and untargeted LC-MS metabolomics studies [1], in the article describing the LC-MS investigation of the changes in the rice metabolome induced by Cd and Cu exposure [26], and finally, in the recent validation of the ROIMCR method for untargeted qualitative and quantitative LC-MS analyses of the lipidomic [27]. All these previous studies have confirmed the suitability of the ROIMCR method in the MS omics data analysis field.

## Conclusions

The chemometric LC-MS data analysis strategy proposed in this study based on the ROIMCR procedure (ROI searching, filtering and compression followed by MCR-ALS analysis) has been shown to be a powerful approach to analyze LC-MS metabolomic datasets. On one hand, the principal benefit of performing the ROI filtering and compression steps is the capacity to minimize the primary dimensions of the data (gigabytes of storage) while preventing any loss of spectral accuracy. On the other hand, the main advantages attributed to the MCR-ALS analysis include: i) the possibility of immediate chemical identification of the metabolites based on the MS information provided in the analysis, ii) the high degree of interpretability of the results, iii) the flexibility in the structure and nature of the datasets that are potentially able to be analyzed and iv) the added value as a preprocessing method that does not require peak modelling or chromatographic alignment for the simultaneous analysis of multiple samples.

## Additional files

Additional file 1: **Table S1.** Coincident ROIs obtained both with our methodology and with centWave algorithm of XCMS package of Work4Metabolomics http://workflow4metabolomics.org/ webpage. The number of ROIs is indicated in this table together with the difference in m/z among these values. (DOCX 26 kb)

Additional file 2: **Figure S1.** LC-MS profiles once imported into MATLAB environment and after ROI compression and filtering and data matrix construction. Example shown for lipid extracts from control brain, gonads and intestinal tract from one simple female Zebrafish. See also Mutagenesis, 2017, 32, 91–103, open access publication at doi:https://doi.org/10.1093/mutage/gew050. (TIF 514 kb)

Additional file 3: **Figure S2.** Augmented LC-MS ROI data matrices of brain samples (Control, C60, SWCNT, ShWCNT and LWCNT) of 15 female and male Zebrafish samples. See also Mutagenesis, 2017, 32, 91–103, open access publication at doi:https://doi.org/10.1093/mutage/gew050. (TIF 711 kb)

Additional file 4: **Figure S3.** Final augmented LC-MS ROI data matrix containing information of the 90 samples analyzed. Input matrix for further MCR-ALS analysis. See also Mutagenesis, 2017, 32, 91–103, open access publication at doi:https://doi.org/10.1093/mutage/gew050. (TIF 351 kb)

Additional file 5: **Figure S4. A)** Output of MCR-ALS analysis of the final augmented data matrix to find purest elution and mass spectra profiles. One hundred fifty components were resolved, explaining 99.2% of data variance. **B)** Example of elution and spectra profiles for component 2 in the 90 samples. See also Mutagenesis, 2017, 32, 91–103, open access publication at doi:https://doi.org/10.1093/mutage/gew050. (TIF 682 kb)

Additional file 6: **Figure S5.** Representation of LC-MS elution profiles of MCR-ALS resolved components corresponding to most representative glycerolipid biomarkers. A) Brain female samples. B) Brain male samples. C) Gonads female samples. D) Gonads male samples. E) Intestinal tract female samples. F) Intestinal tract male samples. (TIF 645 kb)

Additional file 7: **Figure S6.** Representation of LC-MS elution profiles of MCR-ALS resolved components corresponding to most representative glycerophospholipid biomarkers. A) Brain female samples. B) Brain male samples. C) Gonads female samples. d) Gonads male samples. E) Intestinal tract female samples. F) Intestinal tract male samples. (TIF 633 kb)

## Abbreviations

CWT: Continuous wavelet transformations
HPLC: High performance liquid chromatography
LC-MS: Liquid chromatography coupled to mass spectrometry
MCR-ALS: Multivariate Curve Resolution-Alternating Least Squares
MS: Mass spectrometry
ROI: Regions of interest
TBT: Tributyltin
UHPLC: Ultra-high performance liquid chromatography
