# Supplementary material for: ROIMCR: a powerful analysis strategy for LC-MS metabolomic datasets
Source: BMC Bioinformatics. 2019 May 17;20:256. doi: 10.1186/s12859-019-2848-8 (PMC6525397; doi:10.1186/s12859-019-2848-8)
Supplement: Supplementary file 1 — Table S1. Coincident ROIs obtained both with our methodology and with centWave algorithm of XCMS package of Work4Metabolomics http://workflow4metabolomics.org/ webpage. The number of ROIs is indicated in this table together with the difference in m/z among these values. (DOCX 26 kb) [file 12859_2019_2848_MOESM1_ESM.docx]

| **Supplementary Table 1**. Coincident ROIs obtained both with our methodology and with *centWave* algorithm of XCMS package of Work4Metabolomics <http://workflow4metabolomics.org/> webpage. The number of ROIs is indicated in this table together with the difference in *m/z* among these values. | | | | |
| --- | --- | --- | --- | --- |
| **MATLAB home-made routines** | | ***centWave* (XCMS)** | |  |
| **ROI number** | **ROI m/z** | **ROI number** | **ROI m/z** | **Difference in *m/z* (ppm)** |
| 1 | 271.187751 | 1 | 271.187678 | 0.3 |
| 2 | 315.252943 | 4 | 315.252447 | 1.6 |
| 4 | 338.340857 | 5 | 338.341634 | 2.3 |
| 5 | 339.285952 | 6 | 339.28601 | 0.2 |
| 6 | 365.302532 | 7 | 365.302344 | 0.5 |
| 7 | 391.28293 | 8 | 391.282678 | 0.6 |
| 9 | 413.288249 | 9 | 413.293523 | 12.8 |
| 10 | 456.383507 | 11 | 456.382908 | 1.3 |
| 11 | 482.35951 | 12 | 482.359416 | 0.2 |
| 12 | 496.338091 | 13 | 496.338452 | 0.7 |
| 13 | 508.375079 | 14 | 508.374695 | 0.8 |
| 14 | 518.366945 | 15 | 518.367391 | 0.9 |
| 15 | 522.353985 | 16 | 522.35474 | 1.4 |
| 17 | 538.520243 | 19 | 538.520864 | 1.2 |
| 18 | 550.34843 | 20 | 550.348255 | 0.3 |
| 20 | 555.534345 | 21 | 555.533995 | 0.6 |
| 22 | 572.561135 | 22 | 572.559966 | 2.0 |
| 23 | 577.516361 | 23 | 577.515709 | 1.1 |
| 24 | 581.549736 | 24 | 581.549652 | 0.1 |
| 26 | 583.56008 | 25 | 583.564419 | 7.4 |
| 27 | 584.52543 | 26 | 584.525024 | 0.7 |
| 29 | 586.537696 | 27 | 586.540267 | 4.4 |
| 30 | 587.542094 | 28 | 587.541833 | 0.4 |
| 32 | 598.577072 | 31 | 598.578539 | 2.5 |
| 34 | 600.58963 | 32 | 600.591068 | 2.4 |
| 35 | 603.530424 | 33 | 603.529592 | 1.4 |
| 38 | 612.554556 | 36 | 612.555709 | 1.9 |
| 39 | 613.558241 | 37 | 613.559153 | 1.5 |
| 40 | 614.564987 | 38 | 614.569684 | 7.6 |
| 42 | 617.511476 | 39 | 617.512199 | 1.2 |
| 43 | 618.519811 | 40 | 618.514441 | 8.7 |
| 44 | 624.589358 | 41 | 624.590602 | 2.0 |
| 46 | 638.569628 | 43 | 638.569272 | 0.6 |
| 47 | 639.575031 | 44 | 639.574483 | 0.9 |
| 48 | 640.583245 | 45 | 640.585723 | 3.9 |
| 49 | 641.58757 | 46 | 641.588497 | 1.4 |
| 50 | 643.523935 | 48 | 643.524145 | 0.3 |
| 51 | 644.548829 | 49 | 644.553923 | 7.9 |
| 52 | 645.551534 | 50 | 645.539969 | 17.9 |
| 53 | 646.60526 | 51 | 646.612275 | 10.8 |
| 54 | 647.459448 | 52 | 647.460573 | 1.7 |
| 56 | 648.4616 | 53 | 648.460125 | 2.3 |
| 57 | 648.623249 | 54 | 648.625082 | 2.8 |
| 58 | 649.462047 | 55 | 649.463192 | 1.8 |
| 60 | 650.614159 | 56 | 650.607986 | 9.5 |
| 62 | 663.452063 | 57 | 663.451976 | 0.1 |
| 64 | 664.526815 | 58 | 664.526873 | 0.1 |
| 65 | 664.585645 | 59 | 664.58528 | 0.5 |
| 66 | 666.601302 | 60 | 666.601202 | 0.2 |
| 67 | 668.583373 | 61 | 668.57629 | 10.6 |
| 69 | 670.608637 | 62 | 670.608632 | 0.0 |
| 70 | 675.542312 | 63 | 675.542092 | 0.3 |
| 72 | 677.556356 | 65 | 677.560242 | 5.7 |
| 73 | 678.510231 | 66 | 678.506155 | 6.0 |
| 76 | 680.481005 | 67 | 680.478158 | 4.2 |
| 77 | 681.482294 | 68 | 681.481793 | 0.7 |
| 79 | 689.558655 | 70 | 689.55914 | 0.7 |
| 81 | 691.546301 | 71 | 691.547394 | 1.6 |
| 82 | 692.550145 | 72 | 692.556778 | 9.6 |
| 83 | 693.562805 | 75 | 693.564133 | 1.9 |
| 85 | 701.558002 | 78 | 701.558172 | 0.2 |
| 86 | 702.525878 | 79 | 702.534257 | 11.9 |
| 88 | 703.573205 | 80 | 703.57334 | 0.2 |
| 90 | 704.578794 | 81 | 704.573021 | 8.2 |
| 92 | 705.588917 | 82 | 705.589837 | 1.3 |
| 93 | 706.540128 | 83 | 706.539106 | 1.4 |
| 94 | 706.593785 | 84 | 706.59214 | 2.3 |
| 97 | 714.540742 | 85 | 714.541335 | 0.8 |
| 98 | 714.617897 | 86 | 714.618176 | 0.4 |
| 101 | 716.630597 | 87 | 716.633197 | 3.6 |
| 103 | 718.568384 | 88 | 718.57726 | 12.4 |
| 104 | 719.54889 | 89 | 719.541204 | 10.7 |
| 105 | 720.580733 | 91 | 720.5907 | 13.8 |
| 106 | 721.586787 | 93 | 721.593363 | 9.1 |
| 107 | 722.511351 | 94 | 722.512024 | 0.9 |
| 110 | 724.526181 | 95 | 724.526072 | 0.1 |
| 111 | 725.537782 | 96 | 725.528701 | 12.5 |
| 112 | 726.528473 | 97 | 726.532295 | 5.3 |
| 115 | 728.525403 | 98 | 728.520171 | 7.2 |
| 120 | 732.555117 | 99 | 732.549204 | 8.1 |
| 121 | 733.563011 | 101 | 733.557493 | 7.5 |
| 122 | 734.569802 | 102 | 734.570911 | 1.5 |
| 124 | 736.579281 | 105 | 736.579061 | 0.3 |
| 127 | 741.534775 | 106 | 741.540029 | 7.1 |
| 128 | 742.568806 | 107 | 742.571264 | 3.3 |
| 130 | 744.578969 | 109 | 744.572122 | 9.2 |
| 131 | 745.565954 | 110 | 745.557792 | 10.9 |
| 132 | 746.592751 | 112 | 746.603751 | 14.7 |
| 133 | 747.59404 | 114 | 747.603301 | 12.4 |
| 136 | 748.589198 | 115 | 748.581644 | 10.1 |
| 138 | 749.621866 | 117 | 749.627438 | 7.4 |
| 144 | 754.542108 | 118 | 754.538083 | 5.3 |
| 145 | 755.563089 | 120 | 755.573423 | 13.7 |
| 146 | 756.554316 | 121 | 756.550394 | 5.2 |
| 147 | 757.55834 | 122 | 757.55783 | 0.7 |
| 148 | 758.574122 | 123 | 758.570704 | 4.5 |
| 149 | 759.606886 | 125 | 759.61097 | 5.4 |
| 150 | 760.58768 | 127 | 760.585073 | 3.4 |
| 151 | 761.59147 | 128 | 761.590703 | 1.0 |
| 153 | 762.593652 | 129 | 762.59665 | 3.9 |
| 156 | 766.559569 | 130 | 766.568522 | 11.7 |
| 158 | 767.547156 | 131 | 767.542424 | 6.2 |
| 160 | 768.578792 | 133 | 768.588976 | 13.3 |
| 162 | 769.587921 | 134 | 769.598217 | 13.4 |
| 164 | 770.600519 | 135 | 770.601316 | 1.0 |
| 166 | 772.610145 | 136 | 772.619678 | 12.3 |
| 167 | 773.614435 | 138 | 773.623592 | 11.8 |
| 169 | 774.607544 | 139 | 774.597626 | 12.8 |
| 171 | 776.573441 | 140 | 776.56607 | 9.5 |
| 175 | 780.554908 | 142 | 780.553297 | 2.1 |
| 177 | 782.570719 | 143 | 782.56787 | 3.6 |
| 178 | 783.575002 | 144 | 783.571808 | 4.1 |
| 182 | 786.604849 | 145 | 786.601094 | 4.8 |
| 184 | 787.606316 | 146 | 787.603781 | 3.2 |
| 185 | 787.662994 | 147 | 787.661397 | 2.0 |
| 187 | 788.615545 | 148 | 788.613372 | 2.8 |
| 190 | 790.546668 | 149 | 790.538308 | 10.6 |
| 194 | 792.586548 | 150 | 792.58366 | 3.6 |
| 196 | 793.592454 | 151 | 793.592747 | 0.4 |
| 198 | 794.601474 | 152 | 794.60127 | 0.3 |
| 199 | 794.721903 | 153 | 794.721948 | 0.1 |
| 200 | 795.606942 | 154 | 795.610576 | 4.6 |
| 202 | 796.617272 | 155 | 796.61926 | 2.5 |
| 203 | 796.736141 | 156 | 796.737428 | 1.6 |
| 204 | 797.625404 | 157 | 797.623617 | 2.2 |
| 205 | 797.740704 | 158 | 797.741661 | 1.2 |
| 207 | 799.649086 | 159 | 799.660401 | 14.1 |
| 208 | 800.63946 | 160 | 800.612133 | 34.1 |
| 210 | 801.62177 | 163 | 801.61505 | 8.4 |
| 212 | 803.543443 | 164 | 803.543786 | 0.4 |
| 214 | 804.55018 | 165 | 804.553334 | 3.9 |
| 218 | 808.581969 | 166 | 808.583945 | 2.4 |
| 219 | 808.769235 | 167 | 808.772122 | 3.6 |
| 220 | 809.588185 | 168 | 809.5883 | 0.1 |
| 221 | 809.767353 | 169 | 809.777047 | 12.0 |
| 222 | 810.598332 | 170 | 810.598012 | 0.4 |
| 224 | 811.667556 | 171 | 811.670421 | 3.5 |
| 226 | 812.671522 | 172 | 812.669438 | 2.6 |
| 229 | 813.670611 | 173 | 813.680093 | 11.7 |
| 230 | 814.640821 | 174 | 814.634027 | 8.3 |
| 232 | 815.638424 | 175 | 815.636809 | 2.0 |
| 234 | 815.697469 | 176 | 815.697874 | 0.5 |
| 235 | 816.587163 | 177 | 816.588483 | 1.6 |
| 237 | 816.662852 | 178 | 816.671818 | 11.0 |
| 239 | 817.604022 | 179 | 817.592307 | 14.3 |
| 241 | 818.602819 | 180 | 818.604779 | 2.4 |
| 243 | 819.609075 | 181 | 819.607493 | 1.9 |
| 245 | 820.616848 | 183 | 820.616104 | 0.9 |
| 246 | 820.738952 | 184 | 820.739976 | 1.2 |
| 248 | 821.745404 | 185 | 821.749611 | 5.1 |
| 249 | 822.633041 | 186 | 822.633669 | 0.8 |
| 250 | 822.75262 | 187 | 822.756346 | 4.5 |
| 251 | 823.640427 | 188 | 823.636858 | 4.3 |
| 252 | 823.757249 | 189 | 823.759284 | 2.5 |
| 253 | 824.649884 | 190 | 824.652325 | 3.0 |
| 254 | 824.765518 | 191 | 824.768537 | 3.7 |
| 256 | 825.773075 | 192 | 825.770633 | 3.0 |
| 258 | 827.694196 | 193 | 827.706005 | 14.3 |
| 263 | 834.598094 | 194 | 834.599851 | 2.1 |
| 265 | 835.603873 | 196 | 835.603836 | 0.0 |
| 267 | 835.7798 | 198 | 835.792189 | 14.8 |
| 272 | 837.779051 | 199 | 837.772522 | 7.8 |
| 273 | 838.557626 | 200 | 838.558926 | 1.6 |
| 279 | 842.659482 | 201 | 842.659542 | 0.1 |
| 281 | 844.737615 | 203 | 844.737019 | 0.7 |
| 283 | 846.633096 | 204 | 846.632764 | 0.4 |
| 286 | 848.769269 | 205 | 848.773967 | 5.5 |
| 287 | 849.774131 | 206 | 849.776347 | 2.6 |
| 288 | 850.782899 | 207 | 850.784538 | 1.9 |
| 290 | 851.789334 | 208 | 851.792746 | 4.0 |
| 292 | 853.724816 | 209 | 853.726999 | 2.6 |
| 294 | 854.732899 | 210 | 854.726613 | 7.4 |
| 295 | 855.747242 | 211 | 855.73551 | 13.7 |
| 296 | 860.778876 | 212 | 860.76824 | 12.4 |
| 298 | 862.816136 | 213 | 862.820137 | 4.6 |
| 300 | 864.825549 | 214 | 864.834138 | 9.9 |
| 301 | 865.832306 | 216 | 865.83846 | 7.1 |
| 303 | 866.720206 | 217 | 866.721988 | 2.1 |
| 306 | 868.737159 | 218 | 868.737769 | 0.7 |
| 310 | 872.769817 | 219 | 872.76767 | 2.5 |
| 312 | 874.782587 | 220 | 874.78567 | 3.5 |
| 313 | 875.79048 | 221 | 875.789363 | 1.3 |
| 314 | 876.801619 | 222 | 876.805726 | 4.7 |
| 315 | 877.805915 | 223 | 877.808413 | 2.8 |
| 316 | 878.814879 | 224 | 878.818969 | 4.7 |
| 318 | 879.820259 | 225 | 879.822262 | 2.3 |
| 319 | 880.72883 | 226 | 880.721777 | 8.0 |
| 321 | 881.726529 | 227 | 881.722205 | 4.9 |
| 322 | 882.741236 | 228 | 882.730347 | 12.3 |
| 327 | 889.814532 | 230 | 889.80192 | 14.2 |
| 328 | 890.819566 | 234 | 890.817283 | 2.6 |
| 329 | 891.826692 | 235 | 891.817856 | 9.9 |
| 332 | 894.753304 | 236 | 894.755999 | 3.0 |
| 334 | 896.76813 | 237 | 896.766502 | 1.8 |
| 335 | 897.77317 | 238 | 897.772871 | 0.3 |
| 336 | 898.786951 | 239 | 898.795862 | 9.9 |
| 340 | 902.815412 | 240 | 902.818769 | 3.7 |
| 341 | 903.821673 | 241 | 903.822518 | 0.9 |
| 342 | 904.831204 | 242 | 904.828771 | 2.7 |
| 343 | 905.837195 | 243 | 905.84133 | 4.6 |
| 344 | 906.846983 | 244 | 906.847447 | 0.5 |
| 345 | 907.769515 | 245 | 907.776072 | 7.2 |
| 347 | 908.785427 | 246 | 908.780694 | 5.2 |
| 349 | 910.80631 | 247 | 910.807199 | 1.0 |
| 357 | 920.769865 | 248 | 920.767837 | 2.2 |
| 358 | 921.774319 | 249 | 921.772046 | 2.5 |
| 359 | 922.787613 | 250 | 922.793072 | 5.9 |
| 360 | 923.787816 | 251 | 923.789028 | 1.3 |
| 361 | 924.79935 | 252 | 924.796021 | 3.6 |
| 364 | 927.820561 | 253 | 927.815717 | 5.2 |
| 365 | 928.831891 | 254 | 928.837706 | 6.3 |
| 367 | 930.842929 | 255 | 930.845884 | 3.2 |
| 368 | 931.854828 | 256 | 931.860104 | 5.7 |
| 369 | 932.860056 | 257 | 932.858865 | 1.3 |
| 370 | 933.869719 | 258 | 933.863031 | 7.2 |
| 376 | 948.801753 | 259 | 948.807151 | 5.7 |
| 377 | 949.806379 | 260 | 949.80776 | 1.5 |
| 381 | 953.832838 | 261 | 953.832726 | 0.1 |
| 382 | 954.846837 | 262 | 954.850111 | 3.4 |
| 383 | 955.847836 | 263 | 955.853173 | 5.6 |
| 384 | 956.861066 | 264 | 956.859847 | 1.3 |
| 404 | 1184.06555 | 265 | 1184.06619 | 0.5 |
| 405 | 1194.82053 | 266 | 1194.81979 | 0.6 |
| 406 | 1195.82283 | 268 | 1195.82357 | 0.6 |
| 409 | 1208.08675 | 270 | 1208.07638 | 8.6 |
| 411 | 1212.03101 | 271 | 1212.02767 | 2.8 |
| 417 | 1265.05996 | 272 | 1265.06 | 0.0 |
| 420 | 1342.92941 | 273 | 1342.92218 | 5.4 |
| 421 | 1343.93619 | 274 | 1343.92697 | 6.9 |
| 422 | 1409.04882 | 275 | 1409.05057 | 1.2 |
| 423 | 1410.04982 | 276 | 1410.05504 | 3.7 |
| 424 | 1412.0771 | 277 | 1412.07685 | 0.2 |
| 426 | 1452.13648 | 278 | 1452.1402 | 2.6 |
| 428 | 1464.11509 | 279 | 1464.11138 | 2.5 |
| 429 | 1465.11797 | 280 | 1465.11476 | 2.2 |
| 432 | 1469.14593 | 281 | 1469.1422 | 2.5 |
| 436 | 1490.18329 | 282 | 1490.1908 | 5.0 |
| 437 | 1491.18582 | 283 | 1491.19278 | 4.7 |
| 438 | 1492.21389 | 284 | 1492.20842 | 3.7 |
| 439 | 1493.21865 | 285 | 1493.22736 | 5.8 |
| 441 | 1504.17121 | 286 | 1504.17382 | 1.7 |
| 442 | 1506.18447 | 287 | 1506.1857 | 0.8 |
| 444 | 1516.14625 | 288 | 1516.14443 | 1.2 |
| 447 | 1520.18224 | 289 | 1520.19133 | 6.0 |
| 448 | 1521.18688 | 290 | 1521.19484 | 5.2 |
| 463 | 1679.53711 | 291 | 1679.52704 | 6.0 |
| 464 | 1680.54319 | 292 | 1680.53896 | 2.5 |
| 471 | 1732.57693 | 295 | 1732.58702 | 5.8 |
| 475 | 1736.61307 | 296 | 1736.61588 | 1.6 |
