# Supplementary figures and images for: ROIMCR: a powerful analysis strategy for LC-MS metabolomic datasets
Source: BMC Bioinformatics. 2019 May 17;20:256. doi: 10.1186/s12859-019-2848-8 (PMC6525397; doi:10.1186/s12859-019-2848-8)

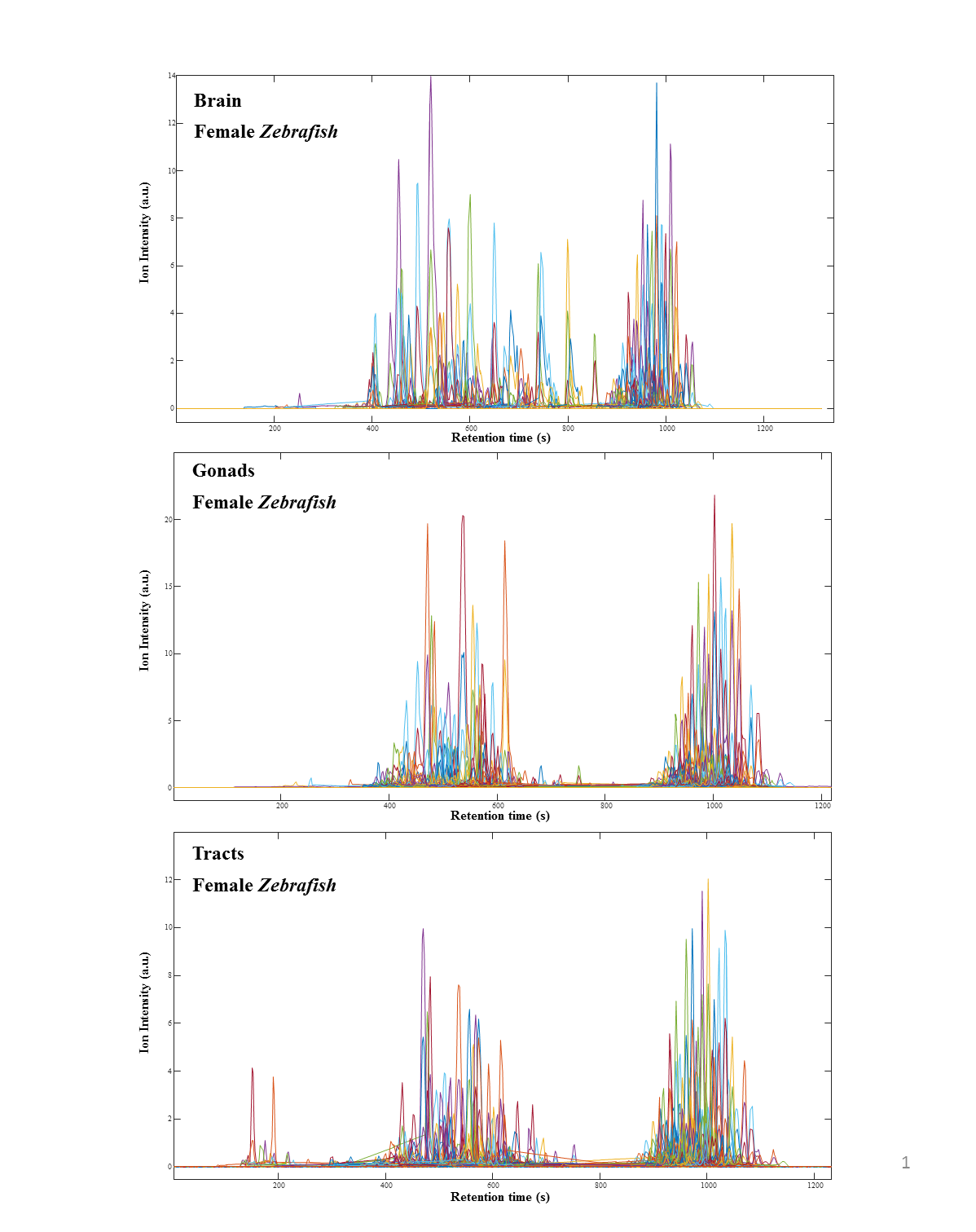

Supplement: Supplementary file 2 — Figure S1. LC-MS profiles once imported into MATLAB environment and after ROI compression and filtering and data matrix construction. Example shown for lipid extracts from control brain, gonads and intestinal tract from one simple female Zebrafish. See also Mutagenesis, 2017, 32, 91–103, open access publication at doi:https://doi.org/10.1093/mutage/gew050. (TIF 514 kb) [file 12859_2019_2848_MOESM2_ESM.tif]

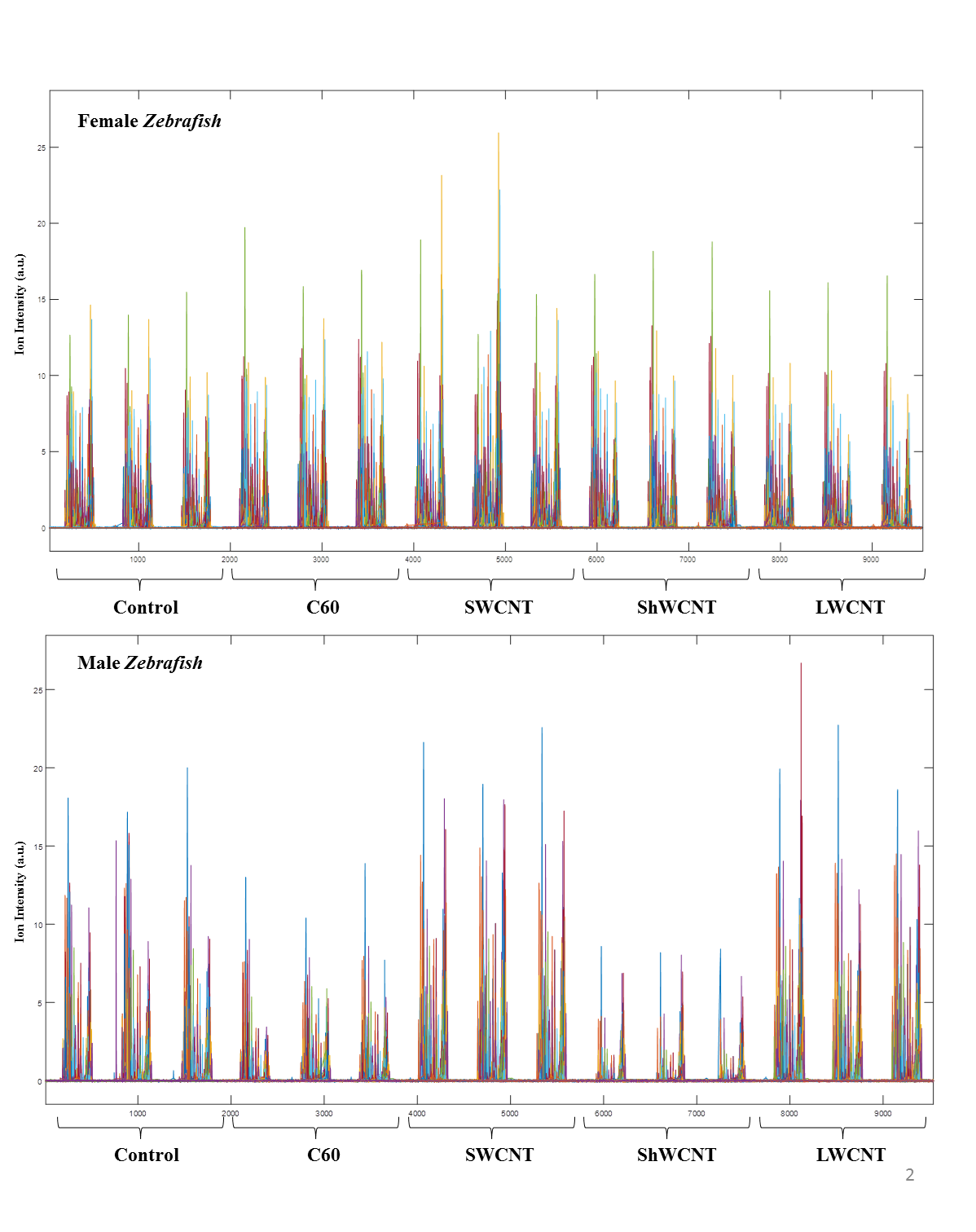

Supplement: Supplementary file 3 — Figure S2. Augmented LC-MS ROI data matrices of brain samples (Control, C60, SWCNT, ShWCNT and LWCNT) of 15 female and male Zebrafish samples. See also Mutagenesis, 2017, 32, 91–103, open access publication at doi:https://doi.org/10.1093/mutage/gew050. (TIF 711 kb) [file 12859_2019_2848_MOESM3_ESM.tif]

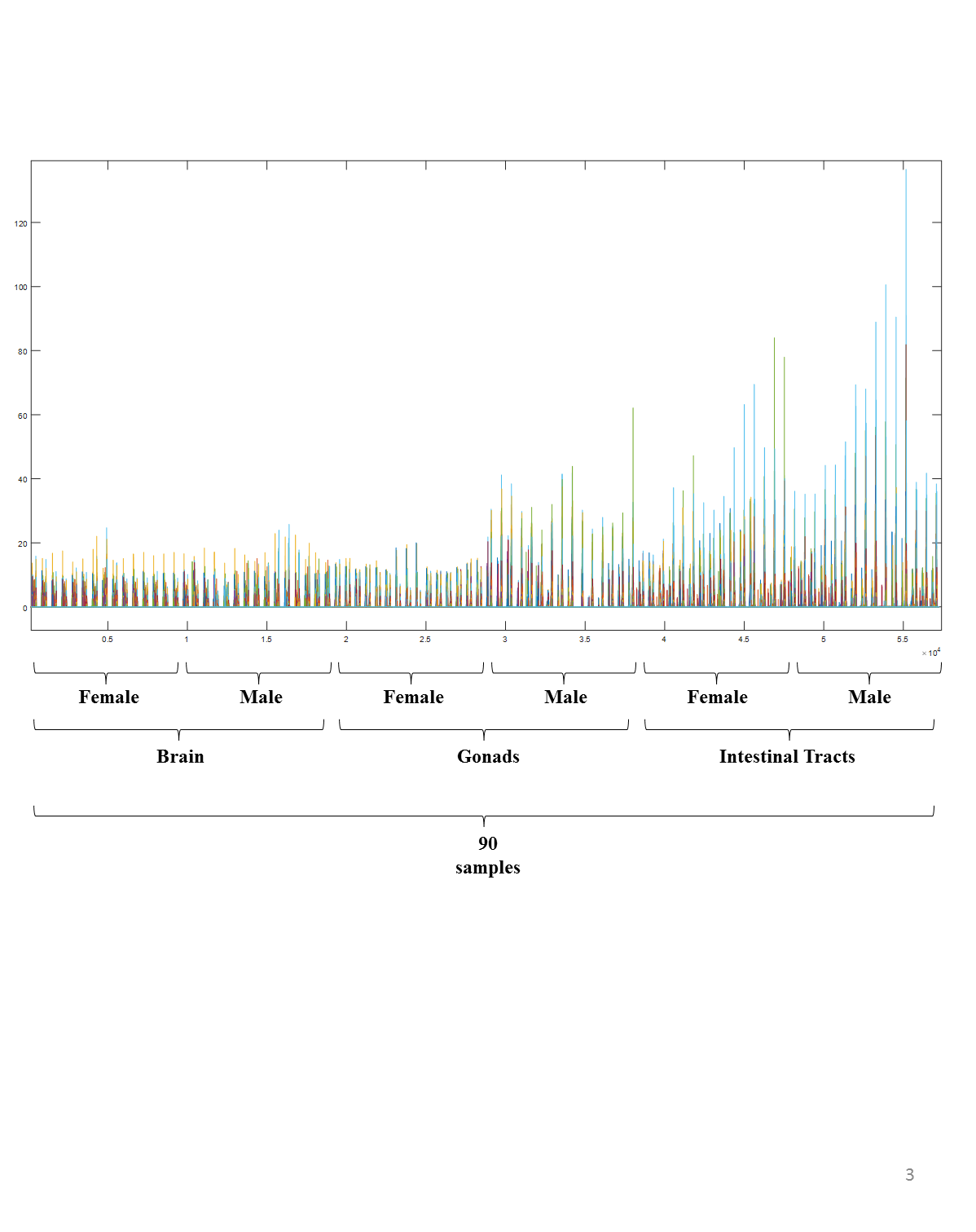

Supplement: Supplementary file 4 — Figure S3. Final augmented LC-MS ROI data matrix containing information of the 90 samples analyzed. Input matrix for further MCR-ALS analysis. See also Mutagenesis, 2017, 32, 91–103, open access publication at doi:https://doi.org/10.1093/mutage/gew050. (TIF 351 kb) [file 12859_2019_2848_MOESM4_ESM.tif]

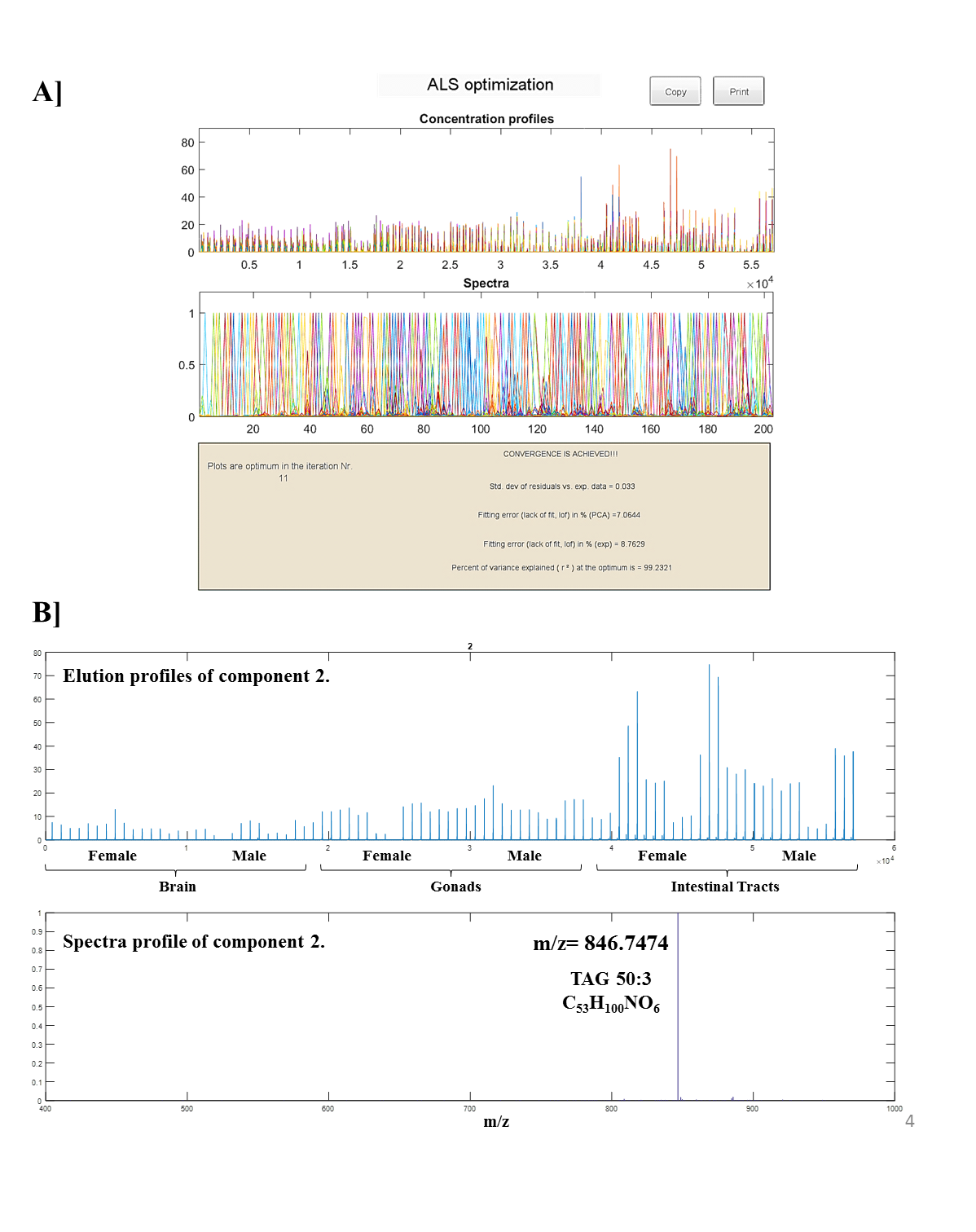

Supplement: Supplementary file 5 — Figure S4. A) Output of MCR-ALS analysis of the final augmented data matrix to find purest elution and mass spectra profiles. One hundred fifty components were resolved, explaining 99.2% of data variance. B) Example of elution and spectra profiles for component 2 in the 90 samples. See also Mutagenesis, 2017, 32, 91–103, open access publication at doi:https://doi.org/10.1093/mutage/gew050. (TIF 682 kb) [file 12859_2019_2848_MOESM5_ESM.tif]

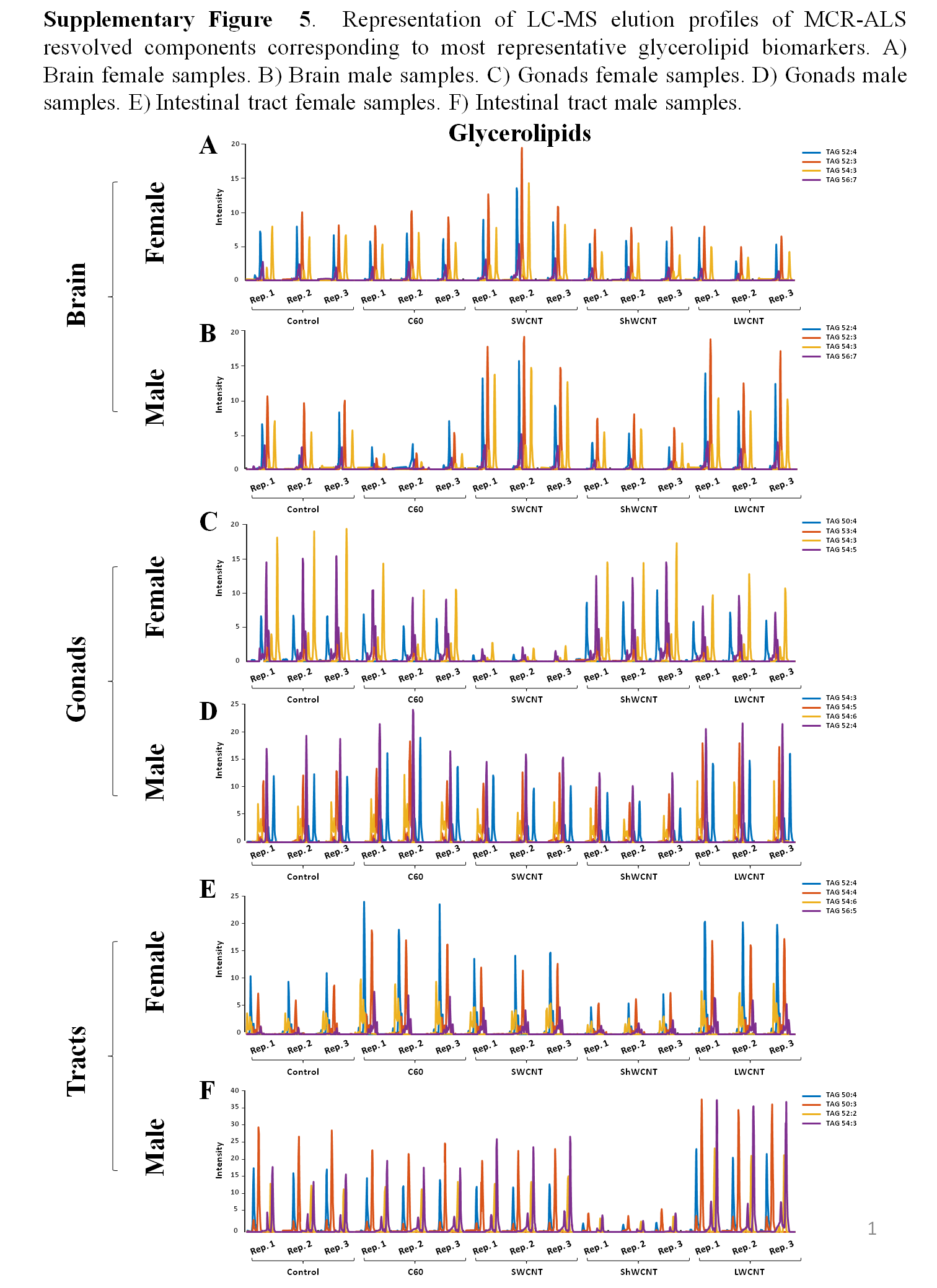

Supplement: Supplementary file 6 — Figure S5. Representation of LC-MS elution profiles of MCR-ALS resolved components corresponding to most representative glycerolipid biomarkers. A) Brain female samples. B) Brain male samples. C) Gonads female samples. D) Gonads male samples. E) Intestinal tract female samples. F) Intestinal tract male samples. (TIF 645 kb) [file 12859_2019_2848_MOESM6_ESM.tif]

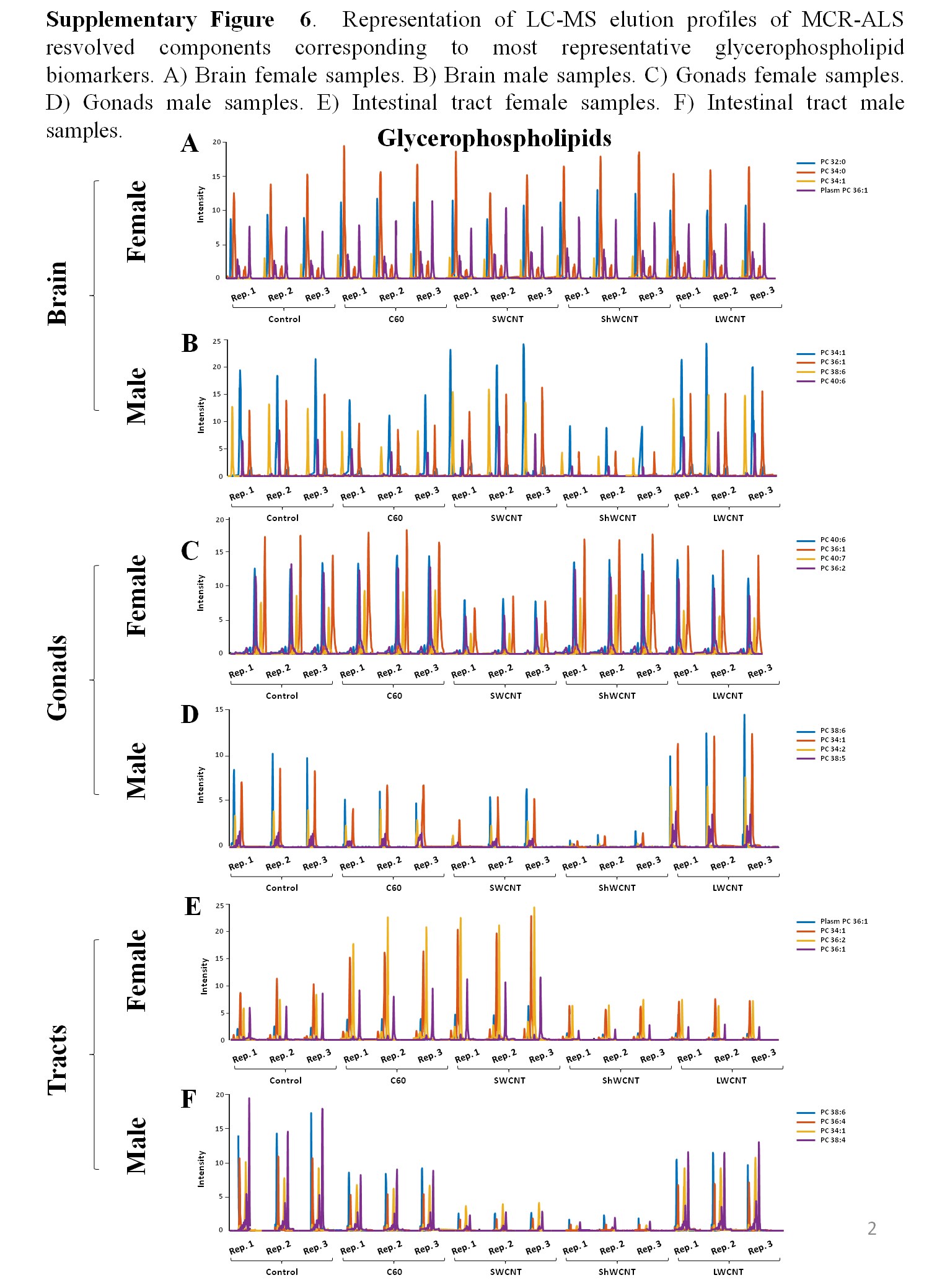

Supplement: Supplementary file 7 — Figure S6. Representation of LC-MS elution profiles of MCR-ALS resolved components corresponding to most representative glycerophospholipid biomarkers. A) Brain female samples. B) Brain male samples. C) Gonads female samples. d) Gonads male samples. E) Intestinal tract female samples. F) Intestinal tract male samples. (TIF 633 kb) [file 12859_2019_2848_MOESM7_ESM.tif]
